# Biological potential of *Lophosoria quadripinnata* fern extract: integration of UHPLC/ESI/QToF/MS analysis, antioxidant activity, molecular docking and molecular dynamics simulation

**DOI:** 10.3389/fphar.2025.1611733

**Published:** 2025-07-11

**Authors:** Alfredo Torres-Benítez, José Erick Ortega-Valencia, Juan Rodrigo Salazar, Jaqueline Ley-Martínez, Javian Gallardo-Valdivia, Marta Sánchez, María Pilar Gómez-Serranillos, Gabriel Vargas-Arana, Mario J. Simirgiotis

**Affiliations:** ^1^ Carrera de Química y Farmacia, Facultad de Ciencias, Universidad San Sebastián, Valdivia, Chile; ^2^ Tecnológico Nacional de México-Instituto Tecnológico Superior de Xalapa, Veracruz, Mexico; ^3^ Departamento de Sistemas Biológicos, División de Ciencias Biológicas y de la Salud, Universidad Autónoma Metropolitana, Mexico City, Mexico; ^4^ Laboratorio Biología de Plantas, Facultad de Ciencias Forestales y Conservación de la Naturaleza, Universidad de Chile, Santiago, Chile; ^5^ Departamento de Farmacología, Farmacognosia y Botánica, Facultad de Farmacia, Universidad Complutense de Madrid, Madrid, Spain; ^6^ Laboratorio de Química de Productos Naturales, Instituto de Investigaciones de la Amazonía Peruana, Iquitos, Peru; ^7^ Facultad de Industrias Alimentarias, Universidad Nacional de la Amazonía Peruana, Iquitos, Peru; ^8^ Instituto de Farmacia, Facultad de Ciencias, Universidad Austral de Chile, Campus Isla Teja, Valdivia, Chile

**Keywords:** fern, lophosoria, extract, metabolites, antioxidant potential, enzymatic inhibition, Nrf2-Keap1 signaling pathway

## Abstract

*Lophosoria quadripinnata* (J.F.Gmel.) C.Chr., a fern species from the Dicksoniaceae family, is widely distributed in Central and South America. This study aimed to identify the bioactive compounds in the aqueous extract of *L. quadripinnata*, evaluate its antioxidant potential through *in vitro* analysis, and assess its neuroprotective effects via molecular docking and dynamics studies. Fourteen compounds were identified using ultra-high-performance liquid chromatography coupled with quadrupole-time-of-flight mass spectrometry (UHPLC-ESI-QToF-MS). *In vitro* assays revealed high concentrations of phenolic and flavonoid compounds, alongside significant antioxidant activity. Molecular docking studies, involving acetylcholinesterase (AChE), butyrylcholinesterase (BChE), tyrosinase, and the Nrf2-Keap1 protein complex, identified three compounds—5C3M (5-O-caffeoyl-3-O-malonylquinic acid), 5GDC (5-O-glucoside-6,7-dimethoxycoumarin), and irifloside—as promising inhibitors. These compounds exhibited favorable binding affinities, minimal toxicity, and strong interactions with key residues involved in the inhibition of the enzymes and protein complex. Additionally, molecular dynamics simulations revealed stable binding with AChE, BChE, and tyrosinase, with irifloside showing the highest binding affinity. The compounds also demonstrated the ability to modulate the Nrf2-Keap1 pathway, potentially enhancing the cellular antioxidant response. These findings suggest that *L. quadripinnata* contains bioactive compounds with significant potential for the development of neuroprotective agents, especially in oxidative stress-related diseases such as Alzheimer’s and Parkinson’s.

## 1 Introduction

Oxidative stress can be defined as a complex biological process in which there is an imbalance between the production of free radicals and the human body’s capacity to eliminate them. Some existing free radicals, which can also be precursors of other free radicals, are reactive oxygen species (ROS), such as peroxyl and hydroxyl radicals. These are the product of metabolic processes involving oxygen, initiating the formation of partially oxidized intermediates with high reactivity. As the human body’s ability to control ROS is insufficient, it can affect various physiological processes, increasing the risk of the onset of diseases such as diabetes, atherosclerosis, myocardial infarction, nervous system disorders, and cancer, among others (Dawidowicz and Olszowy, 2012; Pizzino et al., 2017; Donia and Khamis, 2021; Teleanu et al., 2022; Hajam et al., 2022; Jomova et al., 2023; Selvaraj et al., 2025). Nowadays, products derived from medicinal plants, used due to their therapeutic qualities, have become a raw material not only in research but also an important resource in the pharmaceutical industry (Okaiyeto and Oguntibeju, 2021; Saad, 2023). Plant-derived antioxidants play a crucial role in human health by neutralizing free radicals, reducing oxidative stress, and contributing to cellular protection. These mechanisms are closely associated with aging processes and the prevention of chronic diseases, highlighting their importance in promoting longevity and overall well-being. (Coronado et al., 2015; Alia et al., 2024).

The plant group of ferns contains a wide diversity of species throughout the planet and has been the subject of multiple studies over the years, yielding a wide variety of phytochemical compounds including alkaloids, diterpenes, triterpenes, flavonoids, polyphenols, steroids, among others, which according to studies by characterization of extracts and isolation of particulate compounds and the use of different cellular and animal models, have multiple biological activities (Cao et al., 2017; Pietrak et al., 2022a).

**FIGURE 1:**
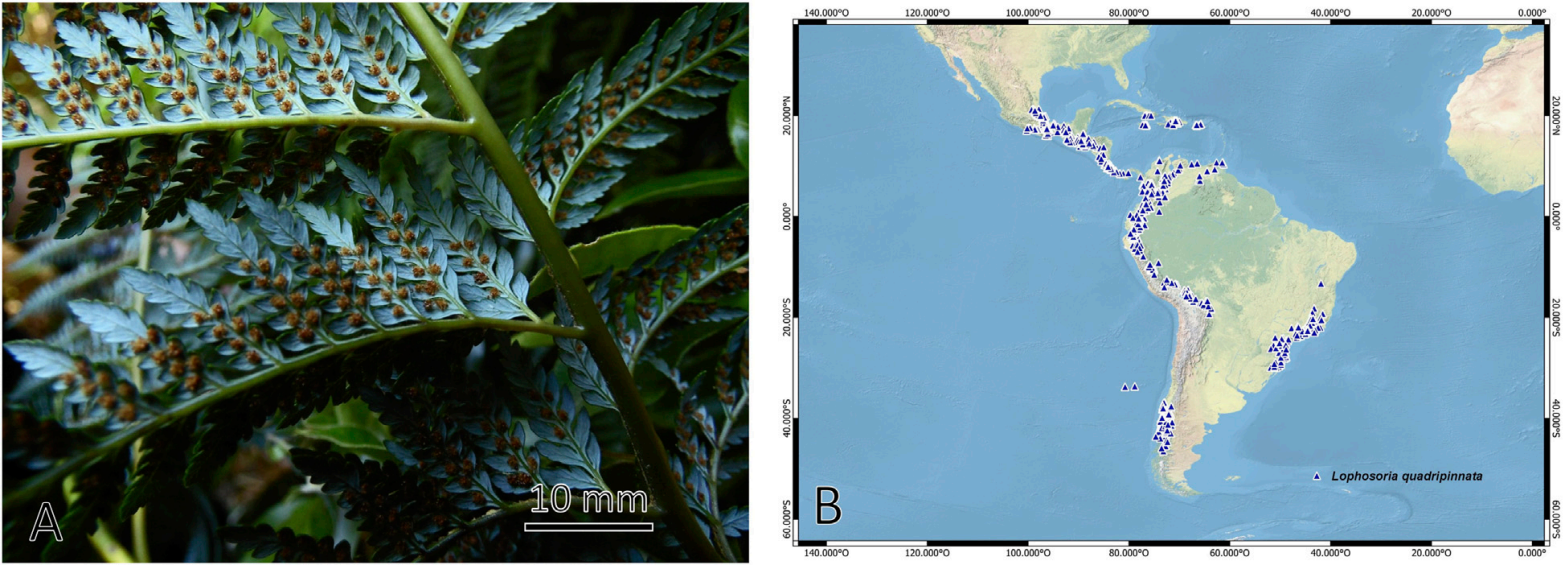
**(A)** Frond; and **(B)** distribution (GBIF) of fern species *Lophosoria quadripinnata*.

The Dicksoniaceae family of the order Cyatheales is made up of plants known as tree ferns that are grouped in the genera *Calochlaena*, *Dicksonia*, and *Lophosoria*, with around 30 species with a mainly Gondwanan distribution (Christenhusz et al., 2011; Noben et al., 2017). The species *Lophosoria quadripinnata* (J.F. Gmel.) C.Chr., known by the common names “ampe”, “palmilla”, “helecho de Valdivia”, “diamondleaf fern”. It is characterized by having a short rhizome or up to 2 m, covered with long hairs; numerous leaves up to 4–5 m long; petioles thick and woody, with long hairs in the juvenile stage and relatively glabrous in the adult stage; leaf blade tripinnate to quadripinnate, with the upper surface dark and shiny green, glabrous and the underside glaucous to whitish; circular sori, 1 mm in diameter and without indusium (Rodriguez et al., 2009; Marquez and Ocampo, 2017) (Figure 1). The difference between this species with the other tree-like ferns of the family Dicksoniaceae lies in the absence of scales on the rhizome and petioles, the absence of indusia, and a glaucous lamina on the underside (http://www.wordlfloraonline.org, accessed on 28 September 2023).

*Lophosoria quadripinnata* is widely distributed in the American tropics and ranges between humid and temperate regions, from Veracruz and Hidalgo in Mexico, El Salvador, Costa Rica, Honduras, Guatemala, Jamaica, Trinidad, Panama, Colombia, Venezuela, Ecuador, Peru, Bolivia, Brazil, to the southern tip of Chile and Argentina (Tryon and Tryon, 1982; Pérez-García et al., 1995). In Chile, it is distributed from the Maule region to the Aysen region and in the Juan Fernandez archipelago, including altitudes from near sea level to 2000 m above sea level. This species develops in humid and shady environments. *Lophosoria quadripinnata* has ornamental value in gardens and as a cut leaf for florists (Rodriguez et al., 2009), also in traditional medicine, the petiole hairs are used as homeostatic and to stop bleeding (Looser and Rodríguez Ríos, 2004) caused by leech bites or small wounds; this benefit is attributed to their secondary metabolites endowed with different bioactivities (Cao et al., 2017), in addition, the use of this fern has been reported to treat hemorrhages and as food in times of scarcity and winter (de Moesbach, 1992). Some metabolites associated with ferns are fatty acids, alkanes, aldehydes, esters, ketones, primary alcohols, phenolic compounds, and terpenoids (Tanaka et al., 1992; Cerón-Carpio et al., 2019).

The objective of this work was to identify the bioactive compounds of the aqueous extract of *L. quadripinnata* fern, to determine its antioxidant capacity by *in vitro* analysis, and neuroprotective effects by the action of major compounds in the inhibition of cholinesterase and tyrosinase enzymes and the activation of the Nrf2-Keap1 protein complex through *in silico* analysis based on docking and molecular dynamics simulations. At the beginning of this research, it was hypothesized that the aqueous extract of *L. quadripinnata* would exhibit significant antioxidant activity and that, based on theoretical studies, some of its compounds could exert neuroprotective effects through interaction with therapeutic targets associated with oxidative stress.

## 2 Materials and methods

### 2.1 Chemicals

Distilled water, ultrapure water, ethyl acetate, ethanol, FeCl_3_, magnesium metal, Folin-Ciocalteu reagent, gallic acid, ascorbic acid, 10% AlCl_3_, CH_3_CO_2_K 1M, quercetin, dimethyl sulfoxide, FeSO_4_, 2,2-diphenyl-1-picrylhydrazyl (DPPH), 2,4,6, tripyridyl-s-triazine (TPTZ), 2,20-azo-bis (2-amidinopropane dichlorohydrate), chlorogenic acid, diosmetin, mexoticin, and daidzein with purity higher than 95% were purchased from Sigma-Aldrich Chem. Co. (St Louis, MO, United States), and analytical grade solvents were obtained from Merck^®^ (Santiago de Chile).

### 2.2 Fern material

Fronds (leaves) of *L. quadripinnata* were collected in an area adjacent to the botanical garden of the Universidad Austral de Chile (Valdivia, Los Ríos region, Chile). Based on a specialized bibliography and the review of morphological characters, was identified by the botanist Javian Gallardo-Valdivia, and a specimen was deposited in the EIF Herbarium of the Faculty of Forestry Sciences and Nature Conservation of the Universidad de Chile (EIF 17430), acronym according to Thiers (continuously updated: http://sweetgum.nybg.org/science/ih/).

### 2.3 Preparation of the aqueous extract

Fronds of *L. quadripinnata* (20 g) were ground manually and mixed with 250 mL of distilled water in ultrasound for 30 min at 40°C, after obtaining a homogeneous solution, the procedure was filtered and repeated three times. The filtered solution was deposited in a FreeZone LABCONCO benchtop lyophilizer; the aqueous material was stored under refrigeration for later use, and a final yield of 9% was obtained.

### 2.4 UHPLC/ESI/QToF/MS analysis

Using an Ultimate 3000 RS UHPLC instrument with Chromeleon 6.8 software, and a Bruker maXis ESI-QToF-MS with Data Analysis 4.0 software, the separation of the phytocompounds present in the aqueous extract of *L. quadripinnata* fern was performed. The extract (5 mg) was dissolved in 2 mL of methanol, filtered with a polytetrafluoroethylene (PTFE) filter and 10 µL were injected into the apparatus. Elution was performed with a binary gradient system with eluent (A) corresponding to 0.1% formic acid in water, and eluent (B) corresponding to 0.1% formic acid in acetonitrile: 1% B isocratic (0–2 min), 1%–5% B (2–3 min), 5% B isocratic (3–5 min), 5%–10% B (5–8 min), 10%–30% B (8–30 min), 30%–95% B (30–38 min) and 1% B isocratic (38–50 min). ESI-QToF-MS experiments were recorded in negative ion mode, and the scan range was between 100 and 12,000 m*/z*. Separation was performed with a Thermo 5 µm C18 80 Å column (150 mm × 4.6 mm) at a flow rate of 1.0 mL/min. Electrospray ionization (ESI) conditions were at a capillary temperature of 200°C, capillary voltage of 2.0 Kv, dry gas flow rate of 8 mL/min and nebulizer pressure of 2 bar, and the experiments were performed in automatic MS/MS mode. The final identification of the compounds was based on mass data, ion fragmentation patterns and comparison with specialized literature.

### 2.5 Quantitative measurements by UHPLC-DAD

An Ultimate 3,000 RS UHPLC-DAD system equipped with Chromeleon 7.2 software was used to measure some of the main compounds in the extract. Peak 3, corresponding to atrovenetin, showed UV maxima at 218 nm and 383 nm; therefore, 383 nm was used for quantification, employing a calibration curve constructed with diosmetin at 383 nm. Peaks 7 and 8, identified as caffeoyl glucose derivatives, and peaks 13 and 14, corresponding to caffeoylquinic acid derivatives, were quantified using a calibration curve of caffeoylquinic acid (chlorogenic acid) at its UV maximum of 320 nm. Peak 10, a coumarin, and peak 11, mexoticin, were quantified using a standard curve prepared with the structurally similar coumarin daphnetin at 320 nm. Peak 12, daidzein, was quantified at its UV maximum of 254 nm. At least six calibration points were used, with concentrations ranging from 5.0 to 125 mg/mL, showing a high correlation coefficient (*R* ^2^ > 0.998) and results are expressed as mean ± standard deviation (SD).

### 2.6 Total phenolic and total flavonoid content

Total phenolic content was determined by the Folin-Ciocalteu method, using 7% Na_2_CO_3_ solution and gallic acid calibration curve; the solutions were incubated for 90 min in the dark and absorbance was measured at 750 nm; the result was expressed as mg of gallic acid per gram of dried fern, and gallic acid was used as the reference compound (Singleton et al., 1999). The determination of total flavonoids was performed with the reaction between the samples and AlCl_3_ in the presence of NaNO_2_ with alkaline medium and quercetin calibration curve (Zhishen et al., 1999); the solutions were incubated, and the absorbance was measured at 510 nm; the result was expressed as mg of quercetin per gram of dried fern, and quercetin was used as the reference compound.

### 2.7 Ferric-reducing antioxidant power (FRAP) assay

It was performed by assaying the reduction of the ferric 2,4,6-tripyridyl-s-triazine complex (TPTZ) 10 mM dissolved in ethanol, using CH_3_COONa-3H_2_O 3.1%/glacial CH_3_COOH 16% dissolved in deionized water, FeCl_3_ 20 mM in 0.02 M HCl aqueous solution, trolox calibration curve and reading the corresponding absorbances of each sample at 593 nm. The result was expressed as micromoles of trolox equivalents per gram of dried fern, and trolox was used as the reference compound (Benzie and Strain, 1996).

### 2.8 Oxygen radical absorbance capacity (ORAC) assay

The compound 2,2′-azobis (2-amidinopropane) dihydrochloride (AAPH) was used in a readout kinetics every 2 minutes for one and a half hours, measuring each solution at an excitation and emission wavelength of 485 and 530 nm respectively. The result was expressed as micromoles of trolox equivalents per gram of dried fern, and trolox was used as the reference compound (Cao and Prior, 1999).

### 2.9 DPPH scavenging activity

A 400 μM solution of 2,2-diphenyl-1-picrylhydrazyl (DPPH) radical in absolute ethanol was used to evaluate antioxidant activity. A gallic acid calibration curve was prepared in the same solvent and used as a standard. The samples were incubated for 30 min, and absorbance was measured at 515 nm. Results were expressed as IC50 values (µg of fern extract/mL), with gallic acid serving as the positive control (Brand-Williams et al., 1995).

### 2.10 Docking simulations

An *in silico* molecular docking was performed to evaluate the binding capacity of *L. quadripinnata* compounds to various crystallographic structures of enzymes and proteins: *Torpedo californica* acetylcholinesterase (TcAChE; PDB ID: 1DX6, resolution 2.30 Å, Greenblatt et al., 1999), human butyrylcholinesterase (hBChE; PDB ID: 4BDS, resolution 2.10 Å, Nachon et al., 2013), tyrosinase from Agaricus bisporus (PDB ID: 2Y9X, resolution 2.78 Å, Ismaya et al., 2011), and human Keap1 (PDB ID: 4L7B, resolution 2.10 Å). All protein structures were obtained from the RCSB PDB database. Optimization of the enzymes was carried out using the UCSF Chimera software (v1.16, San Francisco, California, United States), each of the enzymes had water molecules and ligands removed from the active sites of the enzymes. All polar hydrogen atoms were added at pH = 7.4, and the appropriate ionization states were considered for each of the basic and acidic amino acid residues. The molecular docking box was set to a cube for each of the enzymes with sides 30 Å long (Table 1).

**TABLE 1:** Grid box dimensions and center coordinates used for molecular docking simulations with acetylcholinesterase, butyrylcholinesterase, tyrosinase, and Nrf2-Keap1 using AutoDock Vina.

X	Y	Z	X	Y	Z
Enzymes/Protein	Grid box size (Å)			Grid center coordinate		
Acetylcholinesterase	30	30	30	3.67	65.99	64.08
Butyrylcholinesterase	30	30	30	134.11	115.17	38.13
Nrf2-Keap1	30	30	30	−3.51	2.48	−27.49
Tyrosinase	30	30	30	−0.48	26.95	−43.27

Note: All dockings were performed using 10 independent runs per ligand, with flexible torsions defined according to MMFF94 optimization.

The centroid of the selected residue was chosen based on the putative catalytic site in each of the enzymes, considering its known catalytic amino acids: Ser200 and His440 for acetylcholinesterase (TcAChE) (Castillo et al., 2016; Roy et al., 2019) Ser198 for butyrylcholinesterase (BChE) (Liu et al., 2020a; Amat-ur-Rasool et al., 2022), for Nrf2-Keap1 Tyr334, Ser363, Arg380, Asn414, Arg415, Ser508, Ser555, Tyr572 and Ser602 (Hassanein et al., 2020a) and for tyrosinase Asn81, His244 and His263 (Ismaya et al., 2011; da Silva et al., 2017). The two-dimensional structures of the ligands were processed in ChemDraw 8.0 software (PerkinElmer Informatics, Waltham, MA, United States) where they were saved in. mol format. Subsequently, they were imported into Avogadro software (https://avogadro.cc, accessed April 20, 2022) to perform geometric optimization of each of the reference ligands and inhibitors using the MMFF94 force field function (Torres-Benítez et al., 2022). Molecular docking was performed using each of the respective rigid crystallographic structures of the enzymes/protein (acetylcholinesterase, butyrylcholinesterase, tyrosinase and Nrf2-Keap1) docking them with the flexible ligands (5C3M, 5GDC and irifloside) whose torsion angles were identified (for 10 independent runs per ligand), in addition to the fact that they did not present any toxicological risk and no more than a violation in the Lipinski’s parameters.

To perform the molecular docking, the UCSF Chimera software was used (Volkamer et al., 2012; Chowdhury et al., 2014) where the galantamine compound was used as a reference inhibitor for acetylcholinesterase and butyrylcholinesterase; as a reference inhibitor for Nrf2-Keap1 to the compound (S, R, S) (Hassanein et al., 2020b) and a reference for tyrosinase to the compound kojic acid (Ismaya et al., 2011; da Silva et al., 2017). Polar hydrogens and partial Gasteiger charges were added, and a grid box was created using the Autodock Vina tools at UCSF Chimera. Docking and analysis results were visualized using Discovery Studio Visualizer (BIOVIA, 2023). Once the molecular docking was completed, the best conformation in each of the ligands for hydrogen bonds or π interactions was analyzed, including the binding energy of the free ligand (kcal/mol) (Torres-Benítez et al., 2022). After a successful docking process with the protein, the lowest binding energy for each ligand was determined. The binding site and docked conformations were then visualized to detect interactions. After performing the molecular docking, the inhibition constant was determined for each of the ligands with each of the crystallographic enzymes/protein (Kumar and Sarkar, 2023). The inhibition constant (Ki) was calculated using the following Equation 1:

$$\text{Ki}=e^{\left(∆\text{G}/\text{R}\text{T}\right)}$$ (1)

Where ∆G represents the minimum binding energy of the docked conformations, R is the universal gas constant (R = 1.985 × 10^−3^ kcal mol^−3^K^−3^) and T is the temperature (T = 298.15K) (Kumar and Sarkar, 2023).

To ensure the reliability of the molecular docking protocol, a redocking validation analysis was also performed. The co-crystallized ligands of each protein target—galantamine for acetylcholinesterase (PDB ID: 1DX6) and butyrylcholinesterase (PDB ID: 4BDS), (S, R, S) for the Nrf2-Keap1 complex (PDB ID: 4L7B), and tropolone for tyrosinase (PDB ID: 2Y9X)—were re-docked into their respective active sites using the same docking settings described above. The resulting poses were compared to the crystallographic ligand conformations by calculating the Root Mean Square Deviation (RMSD) values using PyMOL v2.5.0. The docking was considered valid when the RMSD was below the accepted threshold of 2.0 Å. The RMSD values obtained ranged from 0.533 Å to 1.728 Å, confirming high reproducibility of the native poses and validating the accuracy of the docking methodology used in this study.

### 2.11 Calculation of ADME parameters and risk toxicity

To determine the pharmacokinetic properties and toxicological risks of the compounds obtained in *L. quadripinnata*, the Osiris Data Warrior computational tool is used. The molecular descriptors that were calculated are the participation coefficient (cLogP), the number of hydrogen donor bonds (HBD), the number of hydrogen acceptor bonds (HBA), the total number of rotatable bonds (n-ROTB), topological polar surface area (TPSA) and violations of Lipinski’s rule of five. Using the TPSA value, the percentage of absorption (% ABS) was calculated using the following Equation 2 (Torres-Benítez et al., 2022):

$$\%\text{ ABS}=109-\left(0.345\times \text{ TPSA}\right)$$ (2)

For each of the molecules, the possible toxicity risks they could present were evaluated according to their structure and chemical fragments. The toxicity risks that were evaluated were mutagenicity, tumorigenicity, irritation and reproductive effect Torres-Benítez et al., 2022).

### 2.12 Molecular dynamics simulations

To further validate the docking results and assess the stability of the predicted protein-ligand complexes, molecular dynamics (MD) simulations were performed using YASARA Dynamics v.18.4.24 (Yasara GmbH, Vienna, Austria; Krieger and Vriend, 2015) with the AMBER14 force field (Case et al., 2014). Initial structures were derived from the top-scoring docking poses of the multi-target ligands with each protein. Each complex was solvated in a cubic water box (100 Å × 100 Å × 100 Å) with periodic boundary conditions (PBC) to mimic a bulk environment. The system was maintained at 298 K and 0.997 g/cm^3^ water density. Sodium (Na+) and chloride (Cl-) ions were added for charge neutrality, simulating physiological conditions (pH 7.4, 0.9% NaCl). The particle-mesh Ewald (PME) algorithm with a cut-off radius of 8 Å was employed for electrostatic interactions. Simulations were run for 100 ns, recording snapshots every 250 ps with a timestep of 2.5 fs. Equilibrium was established based on Root Mean Square Deviation (RMSD) fluctuations below 2 Å for at least 15 ns. The resulting trajectories were analyzed using YASARA’s built-in tools, including RMSD, Root Mean Square Fluctuation (RMSF), and radius of gyration (Rg) variations over time as well as ligand binding energy (LBE) calculations using the MM-PBSA method. LBE calculations were performed on snapshots from the last 75 ns of the simulations. In Yasara protocols, the more positive the binding energy, the more favorable the interaction (Ahmed et al., 2021). PLIP web tool (Adasme et al., 2021) was used to determine the interactions of final ligand - protein complexes after 100 ns of simulation, and compared with those obtained after the docking process. ProteinsPlus (https://proteins.plus) PoseView was used to create 2D pose depictions of protein - ligand complexes after 100 ns of simulation (Stierand and Rarey, 2010).

### 2.13 Statistical analysis

For each *in vitro* test solution, three measurements were performed, and the results were expressed as the mean ± standard deviation (SD) using Microsoft Excel 2019 (Microsoft Corporation, Redmond, WA, United States). Subsequently, with a one-way analysis of variance with Tukey’s test, statistical significance was corroborated (p < 0.05).

## 3 Results and discussion

### 3.1 Chromatographic analysis of phytocompounds of *L. quadripinnata*

**FIGURE 2:**
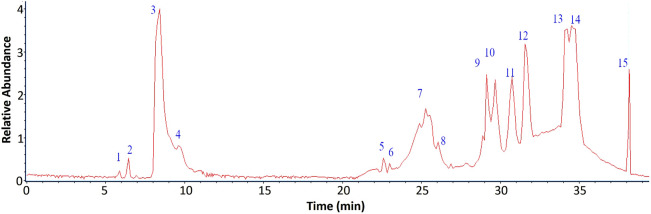
UHPLC-MS Chromatogram at 254 nm of the aqueous extract of *Lophosoria quadripinnata*.

**FIGURE 3:**
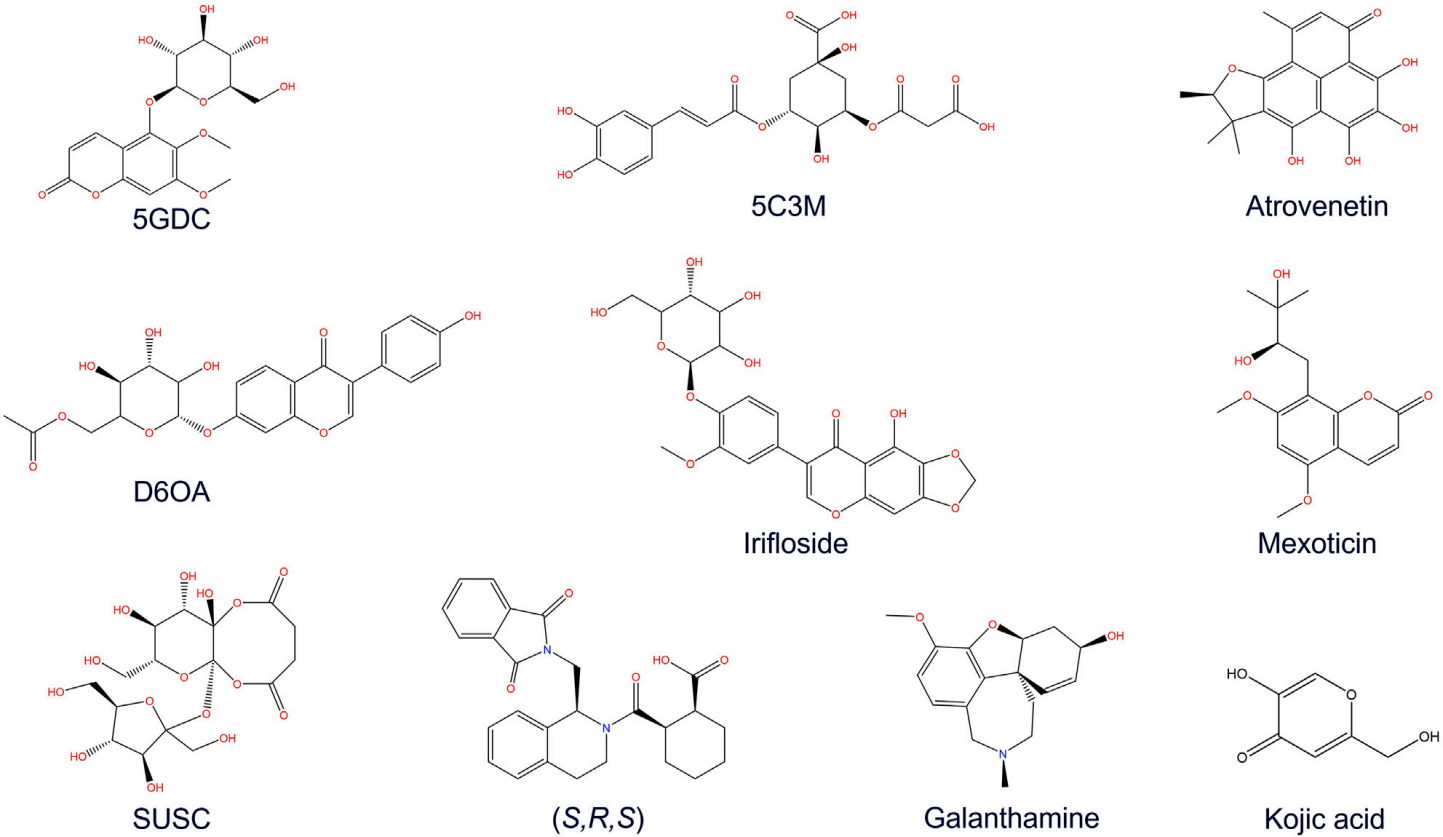
Chemical structures of some major compounds identified in aqueous extract of *Lophosoria quadripinnata*: 5GDC (5-O-glucoside-6,7-dimethoxycoumarin); 5C3M (5-O-caffeoyl-3-O-malonylquinic acid); atrovenetin; D6OA (daidzein 6″-O-acetate); irifloside; mexoticin; SUSC (sucrose succinate); (S, R, S) ((1S, 2R)-2-[(1S)-1-[(1,3-dioxo-2,3-dihydro-1H isoindol-2-yl)methyl]-1,2,3,4-tetrahydroisoquinolin-2-carbonyl]cyclohexane-1-carboxylic acid); and galantamine.

The chemical profile of the aqueous extract of the fern *L. quadripinnata* was obtained by high-performance ultra-high chromatography coupled with mass spectrometry (UHPLC-MS). The LC-MS/MS raw data were processed using Bruker’s DataAnalysis software, which enabled noise reduction, spectral deconvolution, and mass peak visualization. For compound structural characterization, we considered accurate mass data and fragmentation patterns, complemented by searches in digital spectral libraries (MoNA–MassBank of North America, SDBS–Spectral Database for Organic Compounds, and SpectraBase), and relevant scientific literature. Fourteen peaks were tentatively identified in negative ionization mode (Figure 2), corresponding to carbohydrate and aromatic compounds, as shown in Table 2, Figure 3 and Supplementary Figures S1–S6. This ionization method, based on the formation of negatively charged ions, is suitable for the detection of compounds such as phenolic acids and flavonoids, which contain functional groups that are easily deprotonated (Straub and Voyksner, 1993).

**TABLE 2:** Identification of metabolites in *Lophosoria quadripinnata* aqueous extract by UHPLC-ESI-QToF-MS.

Peak	Tentative identification	[M-H]^-^	Retention time (min.)	Theoretical mass (*m/*z)	Measured mass (*m/z*)	Accuracy (ppm)	Metabolite type	MS ions (ppm)
1	Unknown	C_4_H_20_O_14_	5.92	273.95109	272.95478	13.47	-	283.25338, 425.0534
2	Unknown	C_9_H_20_O_13_	9.62	316.93804	316.94226	13.31	-	283.25338, 425.0534
3	Atrovenetin	C_19_H_17_O_6_	8.1	341.09808	341.09719	−2.61	F	191.0513, 683.20954 (2M-H)
4	Atrovenetin isomer	C_19_H_17_O_6_	8.1	341.09863	341.09750	−3.31	F	191.0513, 683.20954 (2M-H)
5	6″-O-acetilgenistin	C_23_H_21_O_11_	22.79	473.11383	473.10894	−10.33	A	265.08486
6	Sucrose succinate	C_16_H_23_O_15_	21.17	455.10424	455.10508	1.84	C	359.08599, 279.10013
7	3-O-Caffeoyl glucose	C_15_H_17_O_9_	25.72	341.07924	341.08193	−7.89	F	327.09700, 281.05861, 191.0513
8	5-O-Caffeoyl glucose	C_15_H_17_O_9_	25.92	341.07929	341.07693	−6.92	F	327.09700, 281.05861, 191.0513
9	Irifloside	C_23_H_21_O_12_	29.34	489.09280	489.09672	8.01	I	447.08263, 341.08066
10	5-O-glucoside-6,7-dimethoxycoumarin	C_17_H_19_O_14_	29.7	447.08330	447.07803	−11.79	F	393.04092, 311.15992, 255.01204
11	Mexoticin	C_16_H_19_O_6_	30.94	307.11627	307.11871	7.94	A	255.22768
12	Daidzein 6″-O-acetate	C_23_H_21_O_10_	31.61	457.01110	457.01402	6.38	A	439.09962, 311.16356
13	5-O-Caffeoyl-3-O-malonylquinic acid	C_19_H_19_O_12_	34.23	439.0823	439.0836	2.96	F	341.1264
14	3-O-Caffeoyl-5-O-malonylquinic acid	C_19_H_19_O_12_	35.26	439.1034	439.1048	3.19	F	341.1264
15	Na formiate (internal standard)	C_4_H_2_O_4_	38.6	112.9829	112.9832	2.65	-	-

Note: A = aromatic derivatives; C = carbohydrate; I = isoflavonoid; F = fenolic acids and phenylpropanoid derivatives.

Phenolic acids and phenylpropanoid derivatives: Peak 3 was tentatively identified as atrovenetin (C_19_H_17_O_6_), with a molecular anion *m/z* 341.09808 and with diagnostic peaks at *m/z* 191.0513 and 683.20954, and peak 4 was tentatively identified as atrovenetin isomer. This isomer was identified based on its matching molecular formula, theoretical mass, and mass fragments with atrovenetin, along with the low differences in measured mass and mass accuracy between the two. However, considering that both compounds share the same retention time, future chromatographic and isolation studies should include a commercial standard and additional spectroscopic data such as nuclear magnetic resonance. Peak 7 and 8 were tentatively identified as 3-O-caffeoyl glucose (C_15_H_17_O_9_), with an [M-H]- ion at *m/z* 341.07924 and diagnostic peaks at *m/z* 327.09700, 281.05861 and 191.0513, and 5-O-caffeoyl glucose (C_15_H_17_O_9_), with an [M-H]^-^ ion at *m/z* 341.07929, respectively. Peak 10 was tentatively identified as 5-O-glucoside-6,7-dimethoxycoumarin (C_17_H_19_O_14_) with a molecular anion *m/z* 447.08330 and with diagnostic peaks at *m/z* 393.04092, 311.15992 and 255.01204. Peaks 13 and 14 were tentatively identified as 5-O-caffeoyl-3-O-malonylquinic acid (C_19_H_19_O_12_) with a molecular anion at m/z 439.0823, and 3-O-caffeoyl-5-O-malonylquinic acid (C_19_H_19_O_12_) with a molecular anion at m/z 439.1034, respectively.

Among the isoflavonoids, peak 9 was tentatively identified as irifloside (C_23_H_21_O_12_), showing a molecular anion at m/z 489.09280 and diagnostic fragments at m/z 447.08263 and 341.08066. For the aromatic derivatives, peak 5 was assigned to 6″-O-acetylgenistin (C_23_H_21_O_11_), peak 11 to mexoticin (C_16_H_19_O_6_), and peak 12 to daidzein 6″-O-acetate (C_23_H_21_O_10_), with a [M-H]^-^ ion at m/z 457.01110 and diagnostic fragments at m/z 439.09962 and 311.16356. Regarding carbohydrates, peak 6 was identified as sucrose succinate (C_16_H_23_O_15_), presenting a molecular anion at m/z 455.10424 and characteristic fragments at m/z 359.08599 and 279.10013. Peaks 1 and 2 were detected but remained unidentified, while peak 15 corresponded to sodium formate (C_4_H_2_O_4_) used as the internal standard, for calibration of the mass spectrometer which improves the precision and accuracy in determining the masses of the compounds present in the extract.

These compounds present in the extract of *L. quadripinnata* are part of the group of phytocompounds also reported in other species from Blechnaceae such as *Parablechnum chilense* (ex *Blechnum chilense*), *Blechnum hastatum*, *Lomariocycas magellanica* (ex *Blechnum magellanicum*), *Austroblechnum penna-marina* (ex *Blechnum penna-marina*), *Blechnopsis orientalis* (ex *Blechnum orientale*), *Parablechnum novae-zelandiae* (ex *Blechnum novae-zelandiae*), *Blechnum occidentale* and *Lomaridium binervatum* (ex *Blechnum binervatum*) (Yusuf, 1994; Fons et al., 2010; Lai et al., 2010; Gasper et al., 2016; Andrade et al., 2017; Waswa et al., 2022; Torres-Benítez et al., 2023).

On the other hand, other triterpenoid-type compounds have been identified and isolated from *L. quadripinnata* such as fern-9 (11)-ene, dryocrassol, adipedatol, diplopterol, and a hydroxymethyl derivative of dryocrassol, which are also reported in fern genera such as *Adiantum*, *Polypodium*, and *Dryopteris* (Tanaka et al., 1992). The presence of the isoflavonoid irifloside should also be highlighted, as it has been widely reported in rhizomes of plants from the Iridaceae family, showing high cytotoxic activity (Qin et al., 2007; Xie et al., 2013; Amin et al., 2021). In short, the study of its chemical composition at the compound level allows validating the traditional use of this species, which in the case of the south of the American continent has been used as a homeostatic remedy (Looser and Rodríguez Ríos, 2004).

In general, the identification of the fourteen compounds by UHPLC-ESI-QToF-MS was focused on qualitative characterization, based on accurate mass values, MS/MS fragmentation patterns, comparison with spectral databases, and specialized literature. However, a semi-quantitative approximation was initially performed from the chromatogram using the relative abundances of the compounds selected for the *in silico* study, yielding a percentage estimation relative to the total of 60.8% for irifloside (peak 9), 58.6% for 5GDC (peak 10), and 89.1% for 5C3M (peak 13).

Additionally, a more formal quantification was carried out, revealing that the total content of phenolic compound peaks analyzed in our *L. quadripinnata* fern extract amounts to 146.783 mg/g of extract. Peaks 10 and 11 were the most abundant compounds (64.271 mg/g), followed by the caffeoyl glucosides (peaks 7 and 8) at 37.283 mg/g, the biologically active compound atrovenetin (peak 3) at 18.038 mg/g, the caffeoylquinic acid derivatives (peaks 13 and 14) at 15.281 mg/g, and the lowest content was observed for daidzein 6″-O-acetate (peak 12), with 11.910 mg/g (Table 3).

**TABLE 3:** Quantitation of some compounds in *Lophosoria quadripinnata* aqueous extract by UHPLD-DAD.

Compounds	Atronetin (mg/g dried fern)	3-O-Caffeoyl glucose 5-O-Caffeoyl glucose (mg/g dried fern)	5-O-glucoside-6,7- dimethoxycoumarin Mexoticin (mg/g dried fern)	Daidzein 6″-O-acetate (mg/g dried fern)	5-O-Caffeoyl-3-O- malonylquinic acid 3-O-Caffeoyl-3-O- malonylquinic acid (mg/g dried fern)
*L. quadripinnata*	18.038 ± 0.023	37.283 ± 0.0821	64.271 ± 0.888	11.910 ± 0.995	15.281 ± 0.090

The absence of commercial standards for the major compounds and the exploratory nature of the study limited the possibility of absolute quantifying the bioactive candidates. As a continuation of this work, we plan to carry out the isolation, purification, and structural elucidation (NMR, IR, UV-Vis) of key constituents such as 5C3M, 5GDC, and irifloside. This will allow for more accurate quantification of their concentrations in the extract and evaluation of their therapeutic potential through *in vitro* and *in vivo* models.

### 3.2 Total phenolic and flavonoid content and antioxidant activity

Table 4 shows the values obtained in the assays of total phenols, total flavonoids, and antioxidant activity for the aqueous extract of *L. quadripinnata*. The result of the phenol (49.029 ± 0.015 mg GAE/g) and flavonoid (91.307 ± 0.059 mg QE/g) contents indicate a medium-high concentration of polyphenolic compounds that trigger significant results in FRAP (2,110.051 ± 0.986 µmol Trolox/g), ORAC (2,631.432 ± 0.998 µmol Trolox/g) and especially DPPH tests with an IC_50_ of 78.137 ± 0.010 μg/mL inferring a high antioxidant potential that would allow the decrease of high concentrations of reactive oxygen species.

**TABLE 4:** Total phenolic (TPC) and flavonoid content (FC) and antioxidant activity (FRAP, ORAC, DPPH) of *Lophosoria quadripinnata* aqueous extract.

Assay	TPC (mg GAE/g)	FC (mg QE/g)	FRAP (µmol Trolox/g)	ORAC (µmol Trolox/g)	DPPH IC_50_ (µg/mL)
*L. quadripinnata*	49.029 ± 0.015 ^a^	91.307 ± 0.059	2,110.051 ± 0.986^a^	2,631.432 ± 0.998^a^	78.137 ± 0.010^a^
Gallic acid^b^	-	-	-	-	2.24 ± 0.04^*^

Note: Values correspond to their means ± SD, of three replicates for each trial. Statistically significant values are marked with.

^a^ According to Tukey’s test (p < 0.05).

^b^ Positive control.

For phenolic compound content, *L. quadripinnata* is similar with the data reported for *A. penna-marina* (88.846 ± 0.020 mg GAE/g) and *P. chilense* (34.078 ± 0.010 mg GAE/g) species and in flavonoids with *A. penna-marina* (128.662 ± 0.065 mg QE/g) (Torres-Benítez et al., 2023). For antioxidant activity in FRAP, ORAC and DPPH assays, *L. quadripinnata* extract shows high potential which is close to the outstanding activity reported in *A. penna-marina* (3,301.847 ± 1.050 μmol Trolox/g, 2,677.519 ± 0.096 μmol Trolox/g and 41.818 ± 0.005 μg/mL, respectively) (Torres-Benítez et al., 2023). On the other hand, the flavonoid content is also comparable with those obtained in *Pyrrosia petiolosa* (80.6 mg/g), *Lepisorus carnosus* (ex *Lemmaphyllum carnosum*) (125.9 mg/g), *Goniophlebium amoenum* (ex *Polypodiodes amoena*) (71.4 mg/g), *Diplopterygium glaucum* (ex *Hicriopteris glauca*) (77.6 mg/g), *Davallodes squamata* (ex *Araiostegia imbricata*) (105.9 mg/g) and *Drynaria coronans* (ex *Pseudodrynaria coronans*) (78.9 mg/g) (Cao et al., 2015). With the Asian species *W. unigemmata* reporting a maximum flavonoid content of 151 ± 11.44 mgQE/g and 768 ± 10.4 mg AAE/g for FRAP, the extract of *L. quadripinnata* maintains comparable ranges, however, its phenolic content and IC_50_ in DPPH results lower compared to 873 ± 6.01 mgGAE/g and 6.07 ± 1.4 μg/mL, respectively in *Woodwardia unigemmata* (Takuli et al., 2020).

The polyphenol and flavonoid content, as well as the antioxidant activity of *L. quadripinnata*, differ significantly from those reported for *Athyrium niponicum* and *Dryopteris erythrosora*. These variations are influenced by abiotic factors such as salinity, sunlight exposure, and environmental stress (Pietrak et al., 2022b). In the case of *D. erythrosora*, flavonoid content and antioxidant activity also vary depending on the type of drying pretreatment, such as shade or heat (Zhang et al., 2019). Overall, the antioxidant potential of ferns highlights their promising applications in pharmaceutical and industrial fields, particularly for the development of photosynthesized nanoparticles with diverse uses and impacts (Fierascu et al., 2021).

### 3.3 ADME and risk toxicity prediction

The pharmacokinetic parameters and toxicity risks of the compounds present in the aqueous extract of *L. quadripinnata* were predicted using Osiris DataWarrior software (Figure 3; Tables 5, 6). Table 5 summarizes the ADME properties of each compound, compared to the reference inhibitors galantamine and the standard inhibitor (S, R, S). The results indicate that none of the tested compounds violated more than one of Lipinski’s rules, which are commonly used to assess drug-likeness. Specifically, 5GDC, atrovenetin, D6OA, mexoticin, SUSC, along with both reference inhibitors, fully complied with Lipinski’s criteria, suggesting potential oral bioavailability (Williams et al., 2022).

**TABLE 5:** Pharmacokinetic properties of compounds in the aqueous extract of *Lophosoria quadripinnata* in comparison with the standard inhibitor galantamine on acetylcholinesterase (TcAChE) and butyrylcholinesterase (BChE), standard inhibitor (S, R, S) on Nrf2-Keap1 and standard inhibitor kojic acid on tyrosinase obtained from Osiris Data Warrior software.

Compound	%ABS^a^	TPSA (Å^2^)^b^	MW^c^	cLogP^d^	HBD^e^	HBA^f^	*n*-ROTB^g^	Violation of Lipinski’s rule
Rule	-	-	<500	≤5	≤5	≤10	≤10	≤1
5C3M	37.19	208.12	440.36	−1.11	6	12	9	1
5GDC	59.27	144.14	384.34	−0.98	4	10	5	0
Atrovenetin	72.01	107.22	342.35	2.65	4	6	0	0
D6OA	56.57	151.98	458.42	0.47	4	10	6	0
Irifloside	49.11	173.60	490.42	0.02	5	12	5	1
Mexoticin	79.60	85.22	308.33	1.36	2	6	5	0
SUSC	25.47	242.13	456.35	−3.82	8	15	5	1
Galantamine*	94.53	41.93	287.35	1.19	1	4	3	0
(S, R, S)*	76.23	94.99	446.50	3.03	1	7	4	0
Kojic Acid*	85.97	66.76	142.11	−1.11	2	4	1	0

Note:

^a^ Percentage of absorption (%ABS).

^b^ Topological polar surface area (TPSA).

^c^ Molecular weight (MW).

^d^ Logarithm of partition coefficient between *n*-octanol and water (cLogP).

^e^ Number of hydrogen bond donors (HBD).

^f^ Number of hydrogen bond acceptors (HBA).

^g^ Number of rotable bonds (*n*-ROTB).

Compound 5C3M = 5-O-caffeoyl-3-O-malonylquinic acid; 5GDC, 5-O-glucoside-6, 7-dimethoxycoumarin; D6OA, Daidzein 6″-O-acetate; SUSC, sucrose succinate; (S, R, S) = (1S, 2R)-2-[(1S)-1-[(1,3-dioxo-2, 3-dihydro-1H, isoindol-2-yl)methyl]-1,2,3,4-tetrahydroisoquinolin-2-carbonyl]cyclohexane-1-carboxylic acid. *Standard inhibitor.

**TABLE 6:** Calculation of toxicity risks of compounds in the aqueous extract of *Lophosoria quadripinnata* in comparison with the standard inhibitor galantamine on acetylcholinesterase (TcAChE) and butyrylcholinesterase (BChE), standard inhibitor (S, R, S) on Nrf2-Keap1 and standard inhibitor kojic acid on tyrosinase obtained from Osiris Data Warrior software.

Compound	Mutagenic	Tumorigenic	Irritant	Reproductive effect
5C3M	None	None	None	None
5GDC	None	None	None	None
Atrovenetin	Low	High	None	High
D6OA	None	High	None	High
Irifloside	None	None	None	None
Mexoticin	None	None	High	None
SUSC	None	None	None	High
Galantamine*	None	None	None	None
(S, R, S)*	None	None	High	None
Kojic acid*	High	High	None	None

Note: Compound 5C3M = 5-O-caffeoyl-3-O-malonylquinic acid; 5GDC, 5-O-glucoside-6, 7-dimethoxycoumarin; D6OA, Daidzein 6″-O-acetate; SUSC, sucrose succinate; (S, R, S) = (1S, 2R)-2-[(1S)-1-[(1,3-dioxo-2, 3-dihydro-1H, isoindol-2-yl)methyl]-1,2,3,4-tetrahydroisoquinolin-2-carbonyl]cyclohexane-1-carboxylic acid. *Standard inhibitor.

However, toxicity predictions in Table 6 revealed that atrovenetin, D6OA, mexoticin, and SUSC posed potential toxicological risks, such as mutagenicity or tumorigenicity. Consequently, these compounds were excluded from subsequent molecular docking simulations. The remaining compounds—5GDC, 5C3M, and irifloside—met the pharmacokinetic criteria and exhibited no predicted toxicity, and were therefore selected as potential inhibitors for crystallographic enzyme/protein targets.

Bioavailability was further analyzed using the Topological Polar Surface Area (TPSA), a parameter that reflects a molecule’s ability to permeate membranes and is inversely related to intestinal absorption and blood-brain barrier penetration (Table 5) (Prasanna and Doerksen, 2009). TPSA values were used to estimate the theoretical absorption percentage (Equation 2), providing insight into how much of each compound could potentially be absorbed. Among the tested compounds, atrovenetin (72.01%) and mexoticin (79.60%) showed the highest predicted absorption, with values comparable to those of the reference inhibitors (Table 5). This suggests good membrane permeability, in line with literature indicating that compounds with TPSA < 140 Å^2^ are more likely to be absorbed orally (Veber et al., 2002).

However, one compound exhibited low predicted absorption, and according to the toxicological analysis, its chemical structure may fragment, potentially generating reactive or unstable metabolites that increase toxicity risk (Table 6; Thompson et al., 2016). This highlights the importance of integrating both pharmacokinetic and toxicity data during early drug discovery stages.

In summary, only compounds that did not violate Lipinski’s rules and showed no predicted toxicity were selected as candidate inhibitors for acetylcholinesterase, butyrylcholinesterase, and Nrf2-Keap1. Accordingly, 5C3M, 5GDC, and irifloside were identified as the most promising compounds and were further evaluated via molecular docking.

Although *in silico* predictions using Osiris DataWarrior provided preliminary insights into the toxicity profiles of the identified compounds, no experimental validation of biological safety was performed. This represents a limitation of the current study, as computational predictions may not fully reflect biological responses under physiological conditions (Wang et al., 2022). Future research should incorporate *in vitro* toxicity assessments in human-derived cell lines such as HepG2, which are widely used for hepatotoxicity and metabolism studies (Godoy et al., 2013). Cytotoxicity assays like MTT or LDH (Fotakis and Timbrell, 2006), genotoxicity evaluations via the comet assay (Collins, 2004), and mitochondrial viability tests such as JC-1 staining (Smiley et al., 1991; Huang et al., 2018) are recommended to confirm the safety and therapeutic relevance of the compounds. These analyses will be essential to validate the *in silico* predictions and support their translational potential.

### 3.4 Molecular docking studies

To evaluate the potential of *L. quadripinnata* compounds as enzyme inhibitors, a consolidated molecular docking analysis was performed using only the most promising candidates. These included the compounds 5C3M, 5GDC, and irifloside, selected based on their compliance with essential pharmacokinetic criteria: none showed more than one violation of Lipinski’s rules, and none presented toxicity risks according to *in silico* predictions (Table 7 and Table 8). These criteria are widely accepted in early-stage drug discovery to increase the likelihood of bioactivity, safety, and oral bioavailability (Veber et al., 2002; Baillie and Jordan, 2015).

**TABLE 7:** Binding energies resulting from molecular docking experiments of the selected compounds in the aqueous extract of *Lophosoria quadripinnata.*

Compounds	Lowest energy of docking TcAChE (Kcal/mol)	Lowest energy of docking BChE (Kcal/mol)	Lowest energy of docking Nrf2-Keap1 (Kcal/mol)	Lowest energy of docking tyrosinase (Kcal/mol)
5C3M	−9.90	−8.90	−10.70	−7.50
5GDC	−8.80	−8.90	−9.10	−7.30
Irifloside	−10.10	−8.30	−10.50	−6.70
Galantamine^a^	−10.20	−8.70	--	--
(S, R, S)^a^	--	--	−11.50	--
Kojic acid^a^	--	--	--	−5.30

Note: Compound 5C3M = 5-O-caffeoyl-3-O-malonylquinic acid; 5GDC, 5-O-glucoside-6, 7-dimethoxycoumarin; (S, R, S) = (1S, 2R)-2-[(1S)-1-[(1,3-dioxo-2, 3-dihydro-1H, isoindol-2-yl)methyl]-1,2,3,4-tetrahydroisoquinolin-2-carbonyl]cyclohexane-1-carboxylic acid.

^a^ Standard inhibitor.

**TABLE 8:** The estimated inhibition constants of the ligands docked with acetylcholinesterase (TcAChE), butyrylcholinesterase (BChE), Nrf2-Keap1 and tyrosinase.

Compounds	Estimated inhibition constant (K_i_) (μM) for acetylcholinesterase (TcAChE)	Estimated inhibition constant (K_i_) (μM) for butyrylcholinesterase (BChE)	Estimated inhibition constant (K_i_) (μM) for Nrf2-Keap1	Estimated inhibition constant (K_i_) (μM) for tyrosinase
5C3M	0.054	0.294	0.014	3.135
5GDC	0.348	0.294	0.210	4.396
Irifloside	0.038	0.811	0.019	12.11
Galantamine^a^	0.032	0.412	--	--
(S, R, S)^a^	--	--	0.003	--
Kojic acid^a^	--	--	--	129.1

Note: Compound 5C3M = 5-O-caffeoyl-3-O-malonylquinic acid; 5GDC, 5-O-glucoside-6, 7-dimethoxycoumarin; (S, R, S) = (1S, 2R)-2-[(1S)-1-[(1,3-dioxo-2, 3-dihydro-1H, isoindol-2-yl)methyl]-1,2,3,4-tetrahydroisoquinolin-2-carbonyl]cyclohexane-1-carboxylic acid.

^a^ Standard inhibitor.

Molecular docking simulations were conducted to assess the interaction of these selected compounds with the active sites of key therapeutic targets: acetylcholinesterase, butyrylcholinesterase, tyrosinase, and the Nrf2-Keap1 protein complex. The analysis focused on identifying binding interactions with amino acid residues known to be critical for enzymatic inhibition, thereby predicting potential efficacy. Figures and tables associated with this section summarize the specific binding residues and affinity scores for each compound.

To ensure analytical coherence and avoid fragmentation, only compounds that met both ADME and toxicity criteria were included in the docking study. While additional compounds from the extract presented interesting chemical structures, they were excluded due to predicted toxicity or poor pharmacokinetic profiles. Including them in the docking simulations would not be justifiable without first addressing their safety risks.

Future studies may consider performing docking analyses for these excluded compounds—such as atrovenetin, D6OA, mexoticin, and SUSC—after chemical modifications, derivatization, or formulation strategies that could mitigate their predicted toxicity. This stepwise approach aligns with rational drug design principles, avoiding premature conclusions based on compounds unlikely to progress to *in vivo* validation stages.

Despite the promising *in silico* and *in vitro* antioxidant findings, an important limitation of this study is the absence of biological validation in cellular or animal models. This lack of validation restricts the physiological extrapolation of the observed results (Godoy et al., 2013; Wang et al., 2022). To fully confirm the neuroprotective potential of the identified compounds, future studies should incorporate *in vitro* assays using neuronal cell lines such as SH-SY5Y or PC12, which are widely used in neutoxicity and neuroprotection models (Xie et al., 2020). These models will allow the evaluation of functional endpoints, including cytotoxicity (e.g., MTT and LDH assays), oxidative stress responses (e.g., DCFH-DA assay), and activation of the Nrf2 pathway via expression of target genes such as HO-1 and NQO1 (Loboda et al., 2016; Zhou et al., 2022). IIn vivo studies may also be warranted to explore pharmacokinetics, biodistribution, and therapeutic efficacy under physiological conditions (Xie et al., 2022). The implementation of these assays in future research will provide critical support to validate the computational predictions and further the translational potential of *L. quadripinnata* compounds in the context of neurodegenerative disease models.

It is important to acknowledge that this study did not include a re-docking validation step to assess the accuracy of the molecular docking protocol. Re-docking of native ligands followed by Root Mean Square Deviation (RMSD) analysis is a widely accepted method for verifying that a docking algorithm can reproduce the experimentally determined binding poses. RMSD values below 2.0 Å are generally considered acceptable for validation purposes (Kurze et al., 2022). Moreover, all docking calculations were conducted using AutoDock Vina, which—despite being efficient and widely used—has known limitations in scoring accuracy and conformational sampling (Pagadala et al., 2017). Therefore, no cross-platform validation was performed. Future studies should address this limitation by including re-docking of co-crystallized ligands, reporting RMSD values to ensure pose reliability, and performing comparative docking using at least one alternative tool such as Glide, GOLD, or MOE. This would enhance the robustness and reproducibility of the results, and help ensure that the docking outcomes are not platform-dependent (Chang et al., 2023). Additionally, combining docking with more accurate scoring functions or machine learning-based re-scoring could further refine the prediction of binding affinities (Cichonska et al., 2021).

#### 3.4.1 Acetylcholinesterase (TcAChE) docking results

**FIGURE 4:**
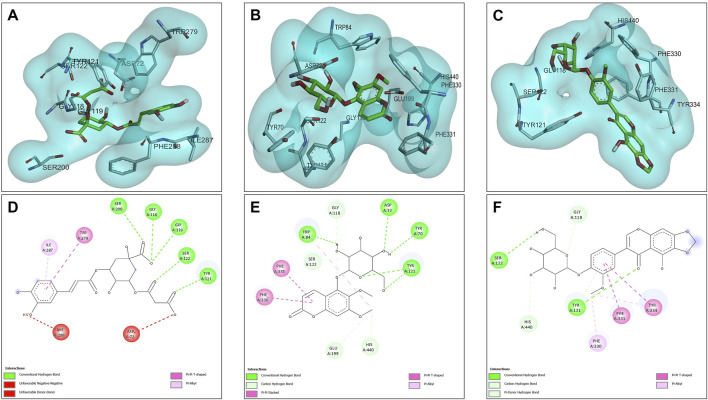
Docking molecular between compounds of *Lophosoria quadripinnata* and the acetylcholinesterase (TcAChE) in a surface view and interactions. **(A–D)** acetylcholinesterase (TcAChE) and 5C3M; **(B–E)** acetylcholinesterase (TcAChE) and 5GDC; and **(C–F)** acetylcholinesterase (TcAChE) and irifloside.

The results of the molecular docking between the candidate acetylcholinesterase inhibitor compounds are shown in Figure 4. This figure shows the interactions of each of the candidates with the acetylcholinesterase residues. Figure 4A,D show the most stable conformation of the 5C3M compound in the catalytic site of acetylcholinesterase. This compound showed 5 hydrogen bond type interactions with the amino acid residues Gly118, Gly119, Tyr121, Ser122, and Ser 200, and the latter residue (Ser200) being directly involved in the inhibition of the acetylcholinesterase enzyme (Sharma, 2019). These 5 hydrogen bond type interactions presented by 5C3M allowed it to present a binding energy (−9.90 kcal/mol) close to that presented by the reference inhibitor (galantamine, −10.20 kcal/mol) (Table 7).

Compound 5GDC was the compound that presented the lowest binding energy (−8.80 kcal/mol) (Figure 4B,E) because it did not present strong interactions (Figure 4E) with the amino acids involved in the stability and inhibition of acetylcholinesterase (Ser200 and His440), however, it is observed that it presented 4 hydrogen bond interactions with residues Tyr70, Asp72, Trp84, and Tyr121; these bonds allowed stability in the molecular docking (Figure 4B,E), however, the efficiency in the inhibition of the enzyme decreases because it does not present direct interactions with the aforementioned residues (Ser200 and His440) in addition to the fact that these results are highly involved in its inhibition values since it was the one that presented the highest values in the inhibition constant (Ki = 0.348) (Table 8).

The compound that presented a slightly higher value in the binding energy with acetylcholinesterase was irifloside (−10.10 kcal/mol) (Table 7). Irifloside presented two hydrogen bonding interactions between a hydroxyl group and the amino acid residue Ser122; and the other hydrogen bond between its carbonyl group of one of its rings and the Tyr121 residue (Figure 4C,F) that gave it important stability in the catalytic site. Pi-donor hydrogen bond type interactions were presented between the imidazole group of residues His440, which is involved in the inhibition of acetylcholinesterase, causing its Ki values (Ki = 0.038) to be similar to that of the reference inhibitor (Ki = 0.032) (Table 8).

#### 3.4.2 Butyrylcholinesterase (BChE) docking results

**FIGURE 5:**
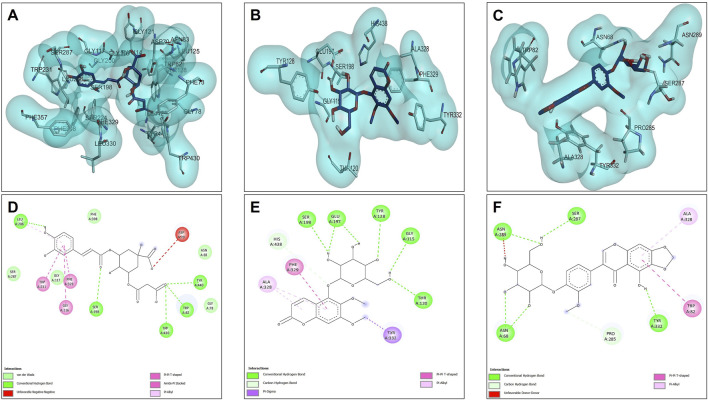
Docking molecular between compounds of *Lophosoria quadripinnata* and the butyrylcholinesterase (BChE) in a surface view and interactions. **(A–D)** butyrylcholinesterase (BChE) and 5C3M; **(B–E)** butyrylcholinesterase (BChE) and 5GDC; and **(C–F)** butyrylcholinesterase (BChE) and irifloside.

The results of the molecular compounds of *L. quadripinnata* and butyrylcholinesterase (BChE) are shown in Figure 5. Compounds 5C3M and 5GDC presented similar binding energies (−8.90 kcal/mol) and inhibition values (Ki = 0.294 for both compound) close to that of the reference inhibitor (galantamine, −8.70 kcal/mol and Ki = 0.412) (Table 7 and Table 8). This behavior is mainly because these two compounds presented ideal stability in the catalytic site of butyrylcholinesterase for the presence in each one of 5 hydrogen bonds with different amino acid residues, one of them being the Ser198 residue that is directly involved in the inhibition and stability of the compounds at the catalytic site (Figure 5A,B,D,E). However, the 5C3M compound may present a little more instability compared to the 5GDC compound because it presented an unfavorable negative-negative interaction between the carboxylate group and the Asp70 residue, which may cause it to have a slight instability compared to the 5C3M compound (Figure 5D).

The compound irifloside was the one with the lowest binding energy compared to the other compounds and the reference inhibitor galantamine. This is mainly because irifloside had 5 hydrogen bonds; two with residues Asn68, one with Ser287, Asn289, and Tyr332; but none of these hydrogen bonds were with residue Ser198, which is highly related to the inhibition of butyrylcholinesterase. However, as shown in Figures 5C,F, these five bonds allow it to have good stability and although the value of union energy (−8.30 kcal/mol) was lower compared to the other compounds (5C3M −8.90, 5GDC −8.90 and galantamine −8.70 kcal/mol) had acceptable performance to be considered as a possible butyrylcholinesterase inhibitor.

#### 3.4.3 Tyrosinase docking results

**FIGURE 6:**
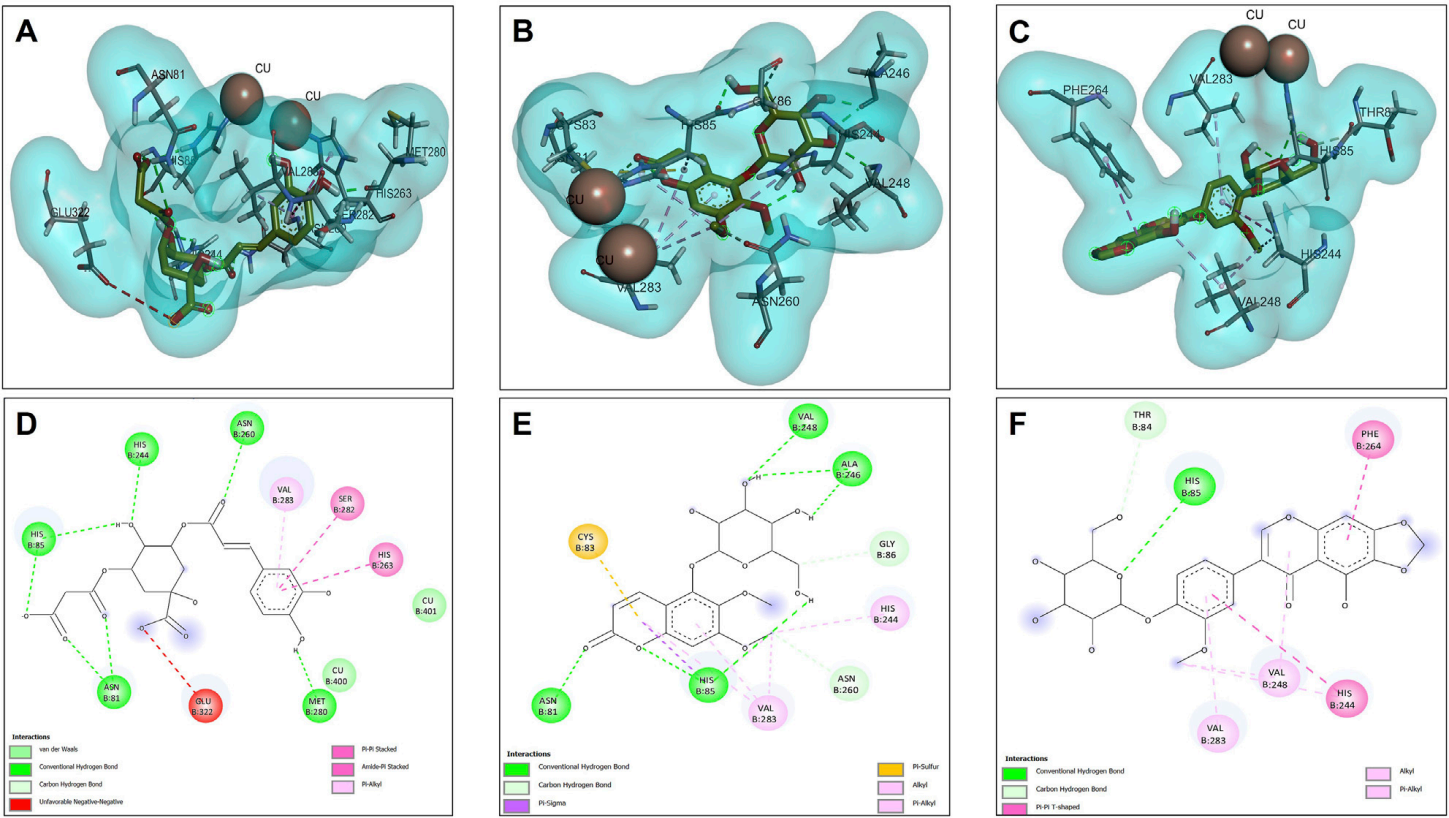
Docking molecular between compounds of *Lophosoria quadripinnata* and the tyrosinase in a surface view and interactions. **(A–D)** tyrosinase and 5C3M; **(B–E)** tyrosinase and 5GDC; **(C–F)** tyrosinase and irifloside.

Molecular docking analyses of the compounds 5C3M, 5GDC and irifloside against the tyrosinase protein (PDB ID: 2Y9X) were performed where the results showed key interactions that may influence their inhibitory potential. The binding affinities for each of these compounds were −7.5 kcal/mol for 5C3M, −7.3 kcal/mol for 5GDC and −6.7 kcal/mol for irifloside. These results, in comparison with the reference inhibitor, kojic acid, which presented a binding affinity of −5.3 kcal/mol, show that the three compounds presented a higher affinity for tyrosinase, suggesting a better inhibitory potential. Figure 6A–C show the three-dimensional representations of the complexes formed between the tyrosinase protein and each of the ligands (5C3M, 5GDC and irifloside), highlighting the most relevant interactions within the active site of tyrosinase. It is observed that the compounds interact with important residues (histidines, polar and hydrophobic amino acids) that are directly involved in the inhibition of tyrosinase and in the stabilization of the complex formed.

Figure 6D shows the two-dimensional interaction map of compound 5C3M, where the formation of 7 hydrogen bonds is observed, which generated significant stability and affinity of compound 5C3M and the active site of tyrosinase. The hydrogen bonds that were present were two with the His85 residue, two with the Asn81 residue, one with the His244 residue, one with the Asn260 residue and one with the Met280 residue. These residues are directly involved in tyrosinase inhibition, showing the great potential of compound 5C3M as a possible tyrosinase inhibitor. Also in this interaction map, three Pi-alkyl interactions were presented between the pi electrons of the aromatic ring of compound 5C3M and the residues His263, Ser282 and Val283. Figure 6E shows the interactions present between compound 5GDC within the active site of tyrosinase. The results showed six hydrogen bond interactions between compound 5GDC and residues Asn81, two interactions with residue His85, two interactions with residue Al246 and one interaction with residue Val248.

These interactions shown by compound 5GDC allowed an important stability in the active site of tyrosinase thus allowing its inhibition. Finally, Figure 6F shows the results of the interactions present in the molecular docking analysis between compound irifloside and tyrosinase. This compound exhibited a behavior similar to the previously evaluated compounds 5C3M and 5GDC, forming a hydrogen bond interaction with the His85 residue. This observation may explain the lower binding affinity of irifloside at the tyrosinase active site, as it formed fewer hydrogen bond interactions compared to the other compounds. However, 2 Pi-Pi interactions with the His244 and Phe264 residues that are directly involved in the inhibition of tyrosinase. These molecular docking results shown by the three compounds were compared with the reference inhibitor (kojic acid). Kojic acid presented the lowest binding energy (−5.3 kcal/mol), which indicates a lower affinity compared to the three compounds analyzed. This suggests that 5C3M, 5GDC and irifloside could be more potent inhibitors, potentially due to their chemical structure that allows for a higher number of interactions and stability within the tyrosinase active site.

The binding energy values obtained suggest that the studied compounds have strong potential to inhibit tyrosinase, even surpassing kojic acid. To further support these findings, molecular dynamics simulations were conducted in addition to the molecular docking analysis, reinforcing the observed behavior of the compounds. This opens the possibility of exploring these candidates in future studies, such as *in vitro* experimental assays, to evaluate their efficacy and stability under biological conditions. Among them, 5C3M, with the highest binding affinity (−7.5 kcal/mol), appears to be the most promising inhibitor due to its strong interactions with key residues and notable stability within the active site.

#### 3.4.4 Nrf2-Keap1 docking results

**FIGURE 7:**
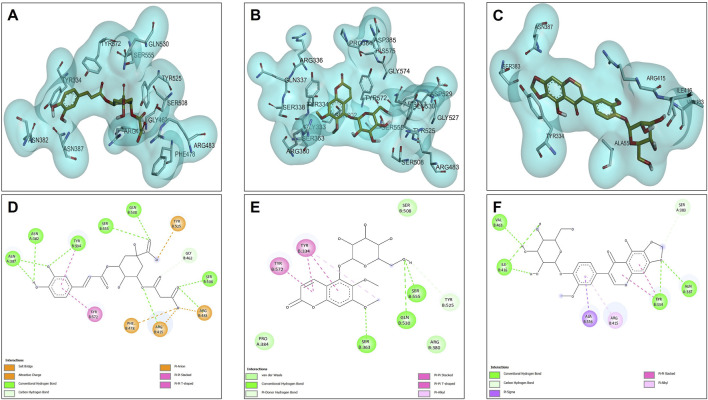
Docking molecular between compounds of *Lophosoria quadripinnata* and the Nrf2-Keap1 in a surface view and interactions. **(A–D)** Nrf2-Keap1 and 5C3M; **(B–E)** Nrf2-Keap1 and 5GDC; and **(C–F)** Nrf2-Keap1 and irifloside.

The opposites 5C3M, 5GDC, and irifloside were evaluated as possible candidate inhibitors of the Nrf2-Keap1 protein. Molecular docking was performed by evaluating their binding affinities in comparison with the reference inhibitor (1S, 2R)-2-[(1S)-1-[(1,3-dioxo-2,3-dihydro-1H isoindol-2-yl) methyl]-1,2,3,4-tetrahydroisoquinolin-2-carbonyl]cyclohexane-1-carboxylic acid (S, R, S). The results showed that the compound 5C3M and irifloside had similar behavior in the binding energy (−10.70 and −10.50 kcal/mol, respectively) (Table 7) with the catalytic site of the Nrf2-Keap1 protein. However, they had lower affinities compared to the reference inhibitor (S, R, S) (−11.50 kcal/mol) (Table 7). The compound 5C3M formed nine hydrogen bonds, including interactions with key residues involved in the inhibition of Nrf2-Keap1 (Tyr334, Arg415, Ser508, Ser555, and Tyr572) (Figure 7A,C), which supports its identification as the most promising candidate for the development of a pharmacological Nrf2-Keap1 inhibitor.

Although the irifloside compound presented a binding energy similar to the 5C3M compound, it is observed that this small difference is mainly because it presented less interaction of hydrogen bonds (5 in total) (Figure 7F), in addition to that, a decrease in the interactions with amino acid residues that are directly involved in inhibition, only observing interactions with residues Tyr334 and Arg415 (Figure 7F). However, these hydrogen bonds give it considerable stability in the binding site, which allows it to be considered as a possible candidate inhibitor of Nrf2-Keap1 (Figure 7C,F). In addition, the molecular interactions of 5C3M with the Nrf2-Keap1 protein are further illustrated in Figure 7D, providing a detailed view of its binding mode and supporting its potential as an effective inhibitor.

The 5GDC compound presented a lower binding energy (−9.10 kcal/mol) compared to the other compounds (−10.70 kcal/mol for 5C3M and −10.50 kcal/mol for irifloside) and with the reference inhibitor (S, R, S) (−11.50 kcal/mol). This behavior is mainly because this compound presented only three hydrogen bond type interactions with residues Ser363, Ser555, and Gln530 where we can observe that residues Ser363 and Ser555 are mainly related to the inhibition of the protein (Figure 7B,E). Although interactions are observed with other amino acid residues involved in the inhibition of the Nrf2-Keap1 protein (Tyr334, Arg380, Ser508, and Tyr572), they do not confer similar stability to the compounds 5C3M and irifloside, which is why it has a lower energy binding (−9.10 kcal/mol) and a lower inhibition constant (Ki = 1.137) compared to the compounds evaluated previously.

The compounds that proved to be optimal in molecular docking with cholinesterase and tyrosinase enzymes and the protein factor Keap1 of the Nrf2-Keap1 complex, demonstrate the multidirectional potential of the secondary metabolites present in ferns and in particular with the species *L. quadripinnata*, to concentrate scientific efforts in the analysis of their effect on *in vitro* and *in vivo* models of oxidative stress and neuroinflammation, which represent pathophysiological events of neurodegenerative diseases such as Parkinson’s and Alzheimer’s (Denzer et al., 2016; Esteras et al., 2016). The antioxidant and enzymatic inhibition analyses of the compounds allow us to filter a relevant activity at the neuroprotective level, and together with the efficacy of binding to Keap1, contribute to the search for the validity of the functions and natural effects of bioactive compounds in the activation of the intracellular signaling pathway of the transcription factor Nrf2, through its accumulation in the cell nucleus for the subsequent activation of the antioxidant and detoxifying defense system (Buendia et al., 2016; Ngo and Duennwald, 2022).

#### 3.4.5 Docking protocol validation by redocking

To reinforce the reliability of the docking methodology employed in this study, a redocking validation was conducted using the crystallographic structures of the enzymes and protein complex under investigation. Specifically, the co-crystallized ligands were redocked into the active sites of acetylcholinesterase (AChE, PDB ID: 1DX6), butyrylcholinesterase (BChE, PDB ID: 4BDS), tyrosinase (PDB ID: 2Y9X), and the Nrf2-Keap1 protein complex (PDB ID: 4L7B). These re-docking experiments aimed to assess whether the docking parameters used could accurately reproduce the experimentally observed ligand conformations (Liu et al., 2020b).

**FIGURE 8:**
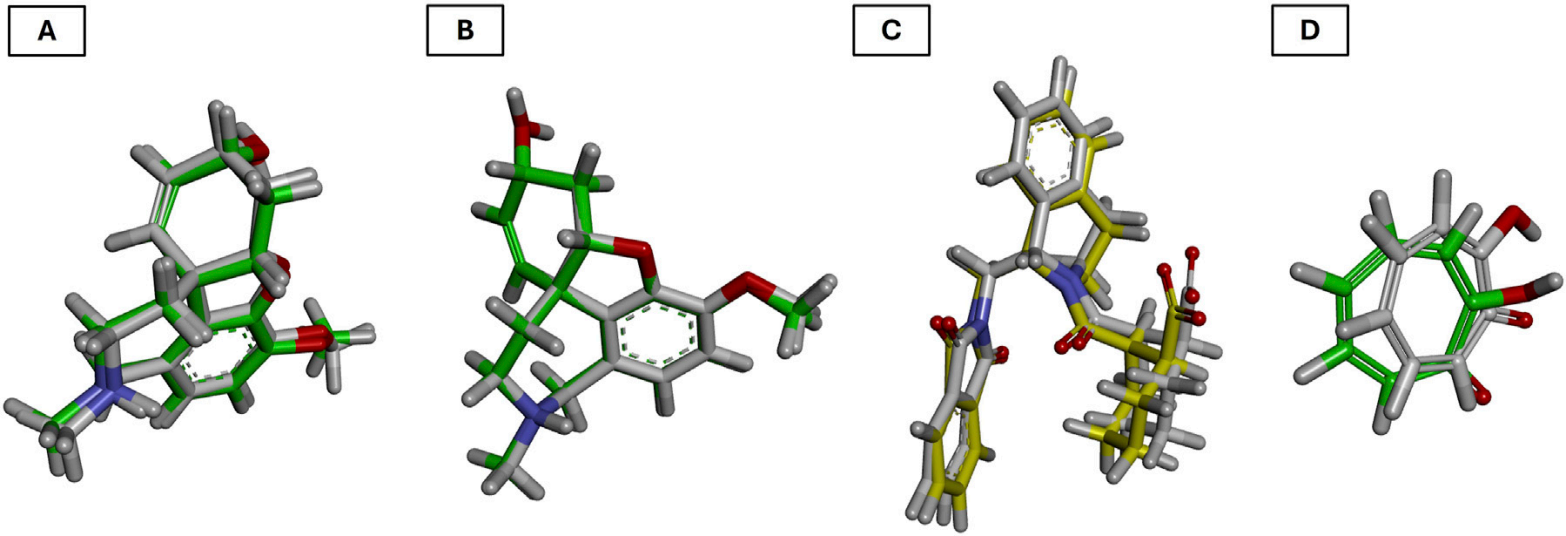
Validation of the docking protocol through redocking of the co-crystallized ligands. **(A)** acetylcholinesterase; **(B)** butyrylcholinesterase; **(C)** Nrf2-Keap1 complex; and **(D)** tyrosinase. Co-crystallized poses in yellow and redocked conformations in green.

For AChE, the redocking of galantamine yielded an RMSD of 0.756 Å and a binding energy of −10.2 kcal/mol (Table 9; Figure 8A). This RMSD is considered excellent, as it is well below the 2.0 Å threshold typically used to validate docking protocols, and even below 1.0 Å, which is indicative of high precision in replicating the crystallographic pose (Ferreira et al., 2015). The alignment of the redocked galantamine with its crystallographic counterpart confirmed the appropriate preparation of both receptor and ligand structures, and the correct definition of the docking grid.

**TABLE 9:** RMSD values (in Å) and binding energies of the self-docking validation for each enzyme-ligand complex.

Protein-PDB	Ligand	Score (Kcal/mol)	RMSD (Å)
Acetylcholinesterase (1DX6)	Galantamine	−10.2	0.756
Butyrylcholinesterase (4BDS)		−8.80	1.728
Nrf2-Keap1 (4L7B)	S, R, S	−11.7	0.533
Tyrosinase (2Y9X)	Tropolone	−6.30	1.550

Note: S, R, S = (1S, 2R)-2-[(1S)-1-[(1,3-dioxo-2, 3-dihydro-1073, 1H isoindol-2-yl)methyl]-1,2,3,4-tetrahydroisoquinolin-2-carbonyl]cyclohexane-1-carboxylic acid.

In the case of BChE, the redocking of galantamine resulted in an RMSD of 1.728 Å and a binding energy of −8.80 kcal/mol (Table 9; Figure 8B). While slightly higher than the AChE result, this RMSD still falls within acceptable limits (Zhang et al., 2017). The observed deviation may be attributed to the greater flexibility of the BChE active site compared to AChE, which is known to influence ligand conformation. Nonetheless, the core alignment of galantamine’s aromatic scaffold and the key hydrogen bonds were preserved.

For the Nrf2-Keap1 complex, the co-crystallized ligand (S, R, S) was redocked into the Keap1 binding site, producing an RMSD of 0.533 Å and a binding energy of −11.7 kcal/mol (Table 9; Figure 8C). This low RMSD reflects excellent accuracy in reproducing the native binding mode and confirms that the docking settings were appropriately configured for this protein-protein interaction domain (Hassanein et al., 2020b).

The redocking of tropolone into the tyrosinase active site resulted in an RMSD of 1.550 Å and a binding energy of −6.30 kcal/mol (Table 9; Figure 8D). Despite the presence of a metal coordination environment and some active site flexibility, the docking protocol effectively recreated the general orientation and interactions of the ligand (Ismaya et al., 2011). The RMSD is within the acceptable range, and deviations are likely due to challenges in modeling metal-ligand interactions precisely (Mugumbate et al., 2014).

Overall, the redocking results for all four targets yielded RMSD values below 2.0 Å, thus validating the docking protocol and supporting the accuracy of the binding poses used in the study. This step not only confirms the structural credibility of the docking predictions but also enhances confidence in the subsequent molecular dynamics simulations and interaction analyses (Wang et al., 2016).

### 3.5 Molecular dynamics

**FIGURE 9:**
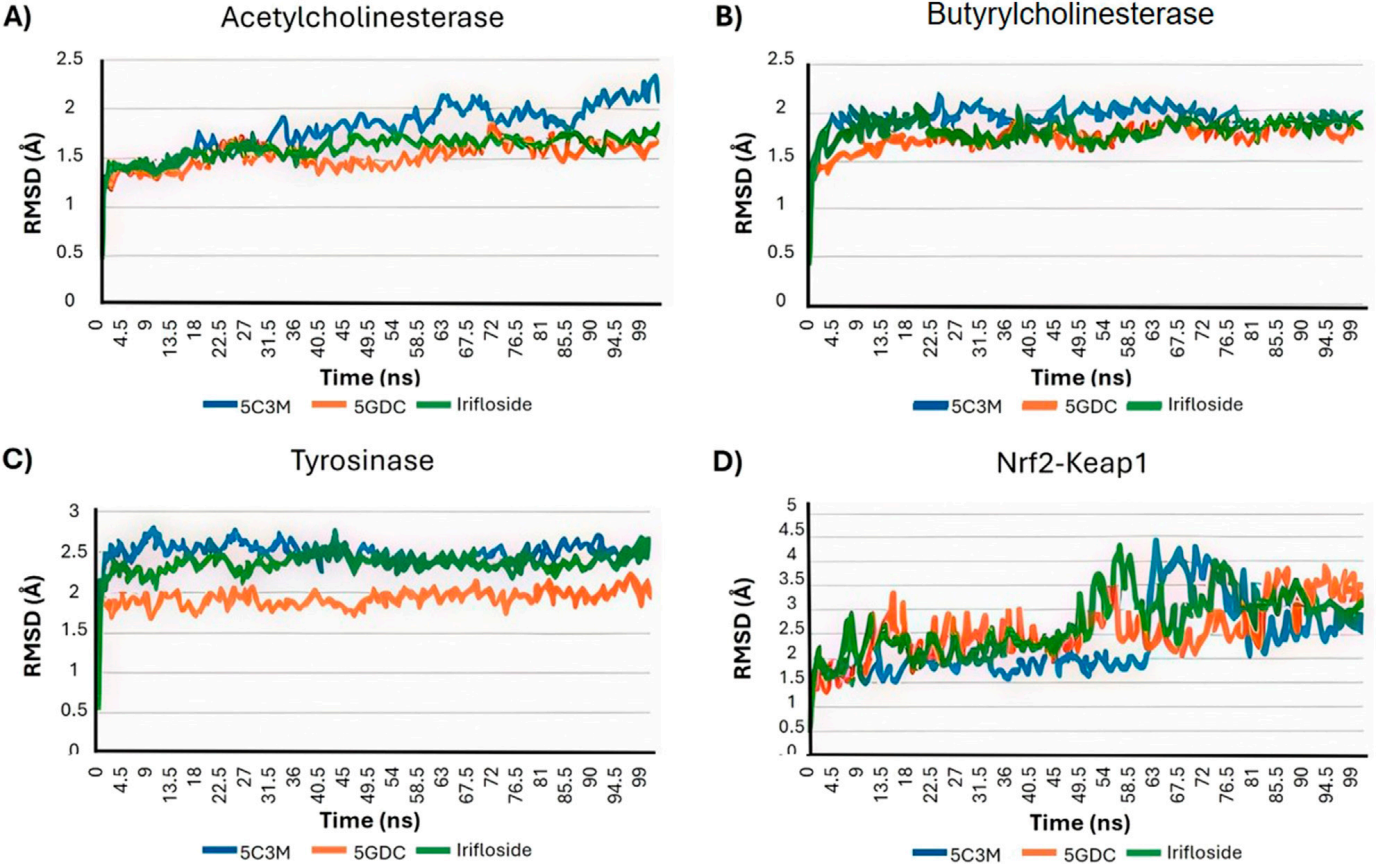
The Root Mean Square Deviation (RMSD in A) analysis plots for the molecular dynamics (MD) simulation trajectories over a 100 ns time scale of 5C3M, 5GDC, and irifloside binded with **(A)** acetylcholinesterase; **(B)** butyrylcholinesterase; **(C)** tyrosinase; and **(D)** Nrf2-Keap1 complex.

The molecular dynamics experiments were carried out using Yasara Software, with 100 ns of simulation. After the simulation time, we wanted to analyze the stability of the complex of 5C3M, 5GDC and irifloside in the Acetylcholinesterase, Butyrylcholinesterase, Tyrosinase and Nrf2-Keap1 and determine their ligand binding energy via Molecular Mechanics Poisson-Boltzmann Surface Area (MM-PBSA) calculations. The set of plots in Figure 9 show the RMSD variations along the simulation time. The ligand binding energies (LBE) of the complexes after stabilization time are shown in Table 10. After a short equilibration time of 15 ns, all complexes were stable, with small changes in the RMSD values, except in Nrf2-Keap1 complexes.

**TABLE 10:** Ligand binding energy (LBE), average RMSD (RMSDA) and average Rg (RgA) of the ligand-protein complexes.

	5C3M			5GDC			Irifloside		
Targets	LBE	RMSD_A_	Rg_A_	LBE	RMSD_A_	Rg_A_	LBE	RMSD_A_	Rg_A_
AchE	−109.217	1.8	23.1	−43.822	1.5	23.0	0.948	1.6	22.9
BChE	−210.711	1.9	23.2	−88.362	1.7	23.1	−9.314	1.8	23.1
Tyrosinase	−209.739	2.5	20.9	18.381	1.9	20.7	0.191	2.4	20.9
Nrf2-Keap1	−196.298	2.3	27.4	−57.103	2.6	27.4	−9.004	2.7	27.3

Note: Compound 5C3M = 5-O-caffeoyl-3-O-malonylquinic acid; 5GDC, 5-O-glucoside-6, 7-dimethoxycoumarin.

#### 3.5.1 Acetylcholinesterase molecular dynamics results

The RMSD plot in Figure 9A evaluates the stability of the protein–ligand complexes. For the 5GDC and irifloside complexes with acetylcholinesterase (AChE), RMSD values remain within 2 Å throughout the 100 ns simulation, indicating the formation of stable complexes. In contrast, the 5C3M complex displays a different behavior. Although the RMSD of the 5C3M ligand appears to stabilize (reaching a plateau) after approximately 30 ns, it begins to gradually increase around 85 ns, reaching 2.5 Å by 100 ns (Figure 9A). This suggests a progressive destabilization of the 5C3M–AChE complex, possibly due to conformational changes in the ligand or disruptions in key ligand–protein interactions.

**FIGURE 10:**
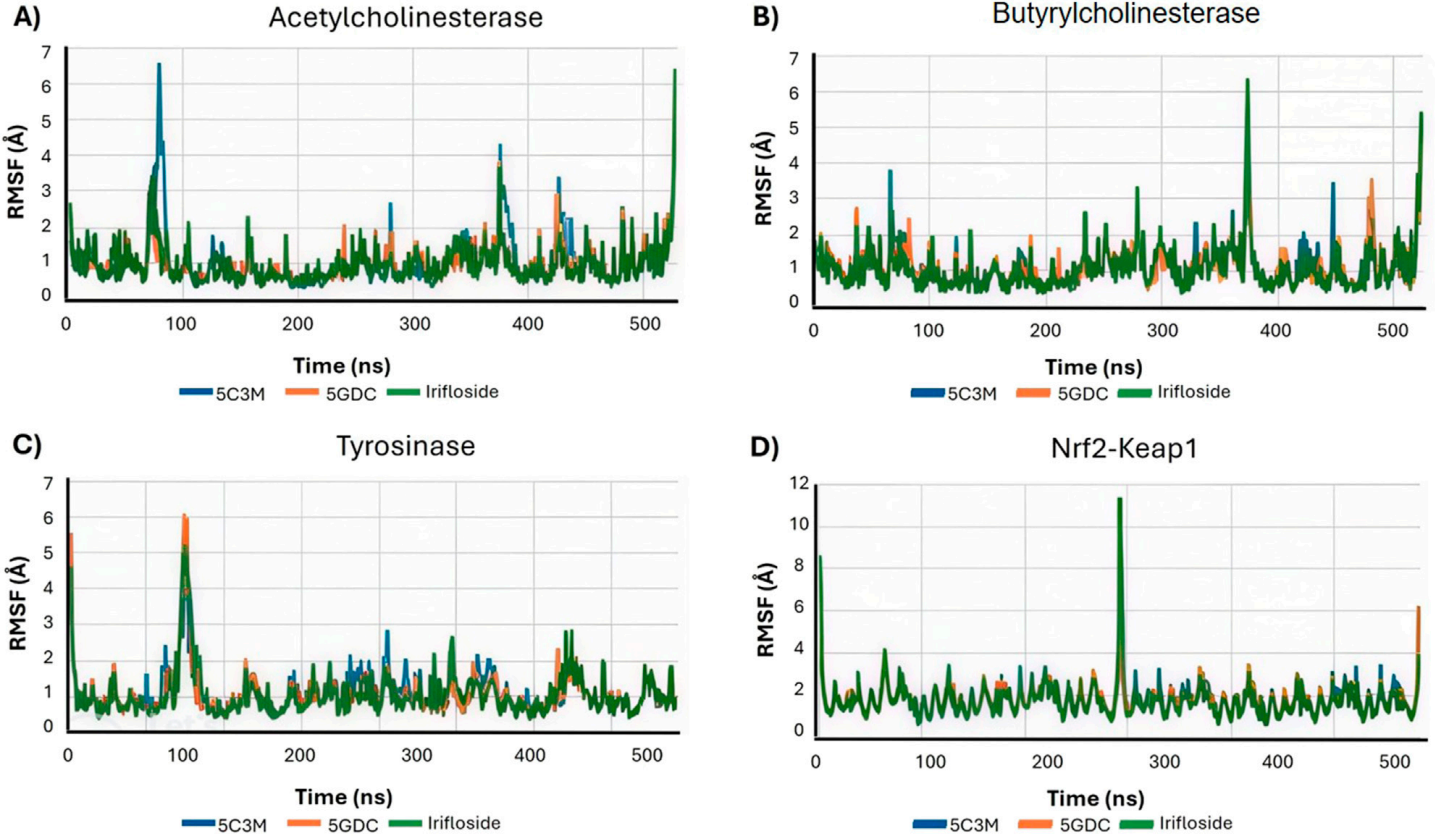
Root Mean Square Fluctuation (RMSF) profiles for each protein-ligand complex after 100 ns of simulation of 5C3M, 5GDC, and irifloside binded with **(A)** acetylcholinesterase; **(B)** butyrylcholinesterase; **(C)** tyrosinase; and **(D)** Nrf2-Keap1 complex.

Figure 10A shows the RMSF profiles for each protein–ligand complex after 100 ns of simulation. A noticeable increase in RMSF values is observed for residues Ser76, Gly77, Ser78, and Glu79 in the 5C3M complex. These elevated fluctuations in this region may contribute to the observed destabilization and could explain the lower binding affinity seen for 5C3M. In contrast, no such fluctuations are detected in the 5GDC and irifloside complexes, suggesting a more stable interaction pattern.

Additionally, analysis of hydrogen bond interactions in the final protein–ligand complexes after 100 ns reveals the loss of key interactions in the 5C3M complex—specifically, the disappearance of a hydrogen bond with Ser200 during the simulation. In the 5GDC complex, the key interaction with Trp84 persists after 100 ns of simulation, indicating its role in maintaining complex stability. Similarly, in the irifloside complex, hydrogen bonds with Ser122 and His440 are retained at the end of the simulation, suggesting that these interactions contribute to the stability of the complex and the potential bioactivity of irifloside. The binding modes and specific interactions supporting these findings are depicted in Figures 12A,B, which visually confirm the presence or loss of critical hydrogen bonds and hydrophobic contacts discussed above.

**FIGURE 11:**
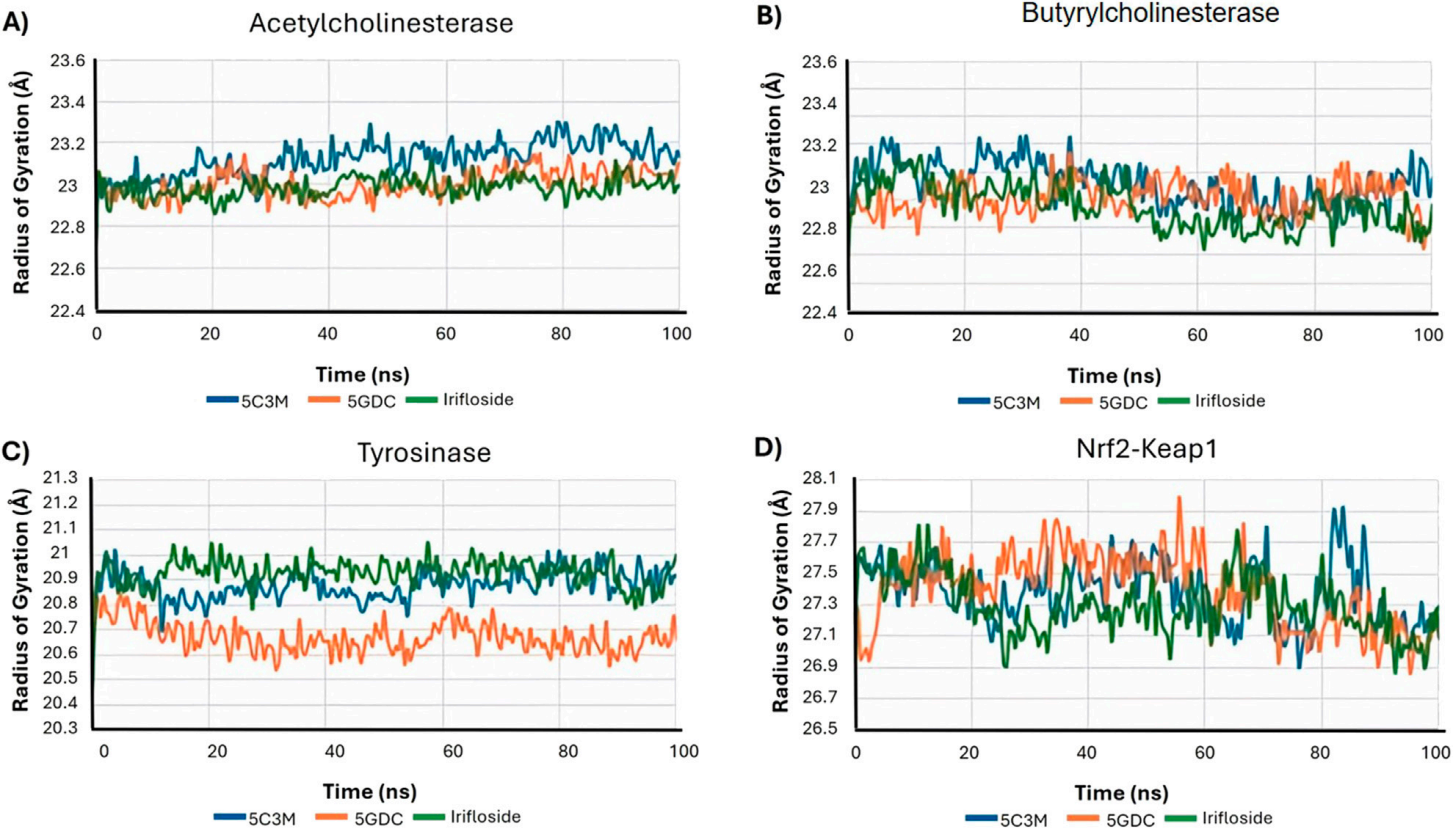
Radius of gyration analysis plots for the molecular dynamics (MD) simulation trajectories over a 100 ns time scale of 5C3M, 5GDC, and irifloside binded with **(A)** acetylcholinesterase; **(B)** butyrylcholinesterase; **(C)** tyrosinase; and **(D)** Nrf2-Keap1 complex.

The radius of gyration (Rg), a measure of the overall compactness of the protein, is presented in Figure 11A. The Rg values for all AChE complexes remain relatively stable throughout the simulation, indicating that the ligands do not cause significant alterations to the protein’s global compactness or overall structure. This supports the idea that the destabilization observed in the 5C3M complex is likely a localized effect rather than a global structural change.

**FIGURE 12:**
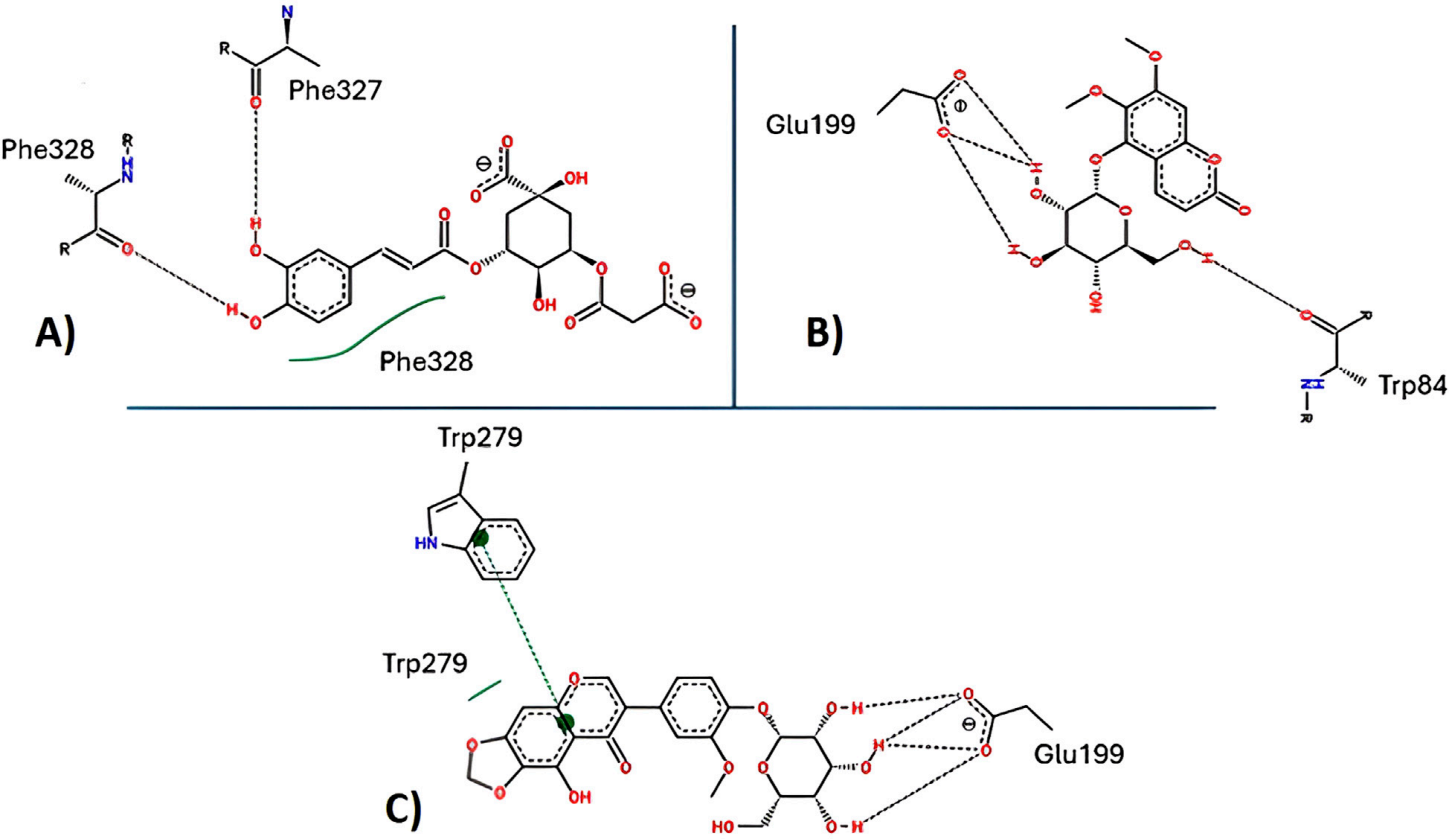
Key interactions between **(A)** 5C3M; **(B)** 5GDC; and **(C)** irifloside with acetylcholinesterase.

Irifloside exhibits a positive LBE value of 0.95 (Table 10), indicating the highest theoretical binding affinity for AChE, which is consistent with the docking study results. This strong binding affinity can be attributed to stable interactions between the aromatic core of irifloside (rings A and C of the isoflavone) and Trp279 in the peripheral anionic site (PAS) of AChE (Figure 12C). Additional stabilizing interactions include hydrogen bonds with His440 in the catalytic triad and Tyr130 in the anionic site, as well as hydrophobic interactions with Phe331 in the anionic site and Tyr334 in the PAS. The combination of these interactions accounts for the high theoretical binding affinity and stability of the irifloside–AChE complex.

#### 3.5.2 Butyrylcholinesterase molecular dynamics results

5C3M, 5GDC, and irifloside each form complexes with butyrylcholinesterase (BChE), as demonstrated by the consistent RMSD, RMSF, and radius of gyration values observed throughout the molecular dynamics simulations (Figure 9B, Figure 10B, Figure 11B, respectively). These plots indicate minimal structural fluctuations and stable compactness over the simulation time of all complexes. The RMSD in Figure 9B shows that the complexes of 5GDC and irifloside remain stable, with minimal fluctuations, indicating robust interactions with BChE. In contrast, while the 5C3M complex also forms a stable complex, any slight increase in RMSD could indicate subtle conformational changes in the ligand over time. Figure 10B shows that there are no significant fluctuations in the RMSF profiles for 5GDC and irifloside, indicating that these complexes remain stable. However, the 5C3M complex exhibits increased fluctuations, particularly in residues Ser76, Gly77, Ser78, and Glu79, which may contribute to the destabilization observed. Figure 11B shows that the radius of gyration (Rg) values remain constant for all complexes, indicating that the ligands do not induce significant changes in the overall compactness of the protein. This suggests that the observed destabilization of the 5C3M complex is likely a localized effect rather than a global change in protein structure.

**FIGURE 13:**
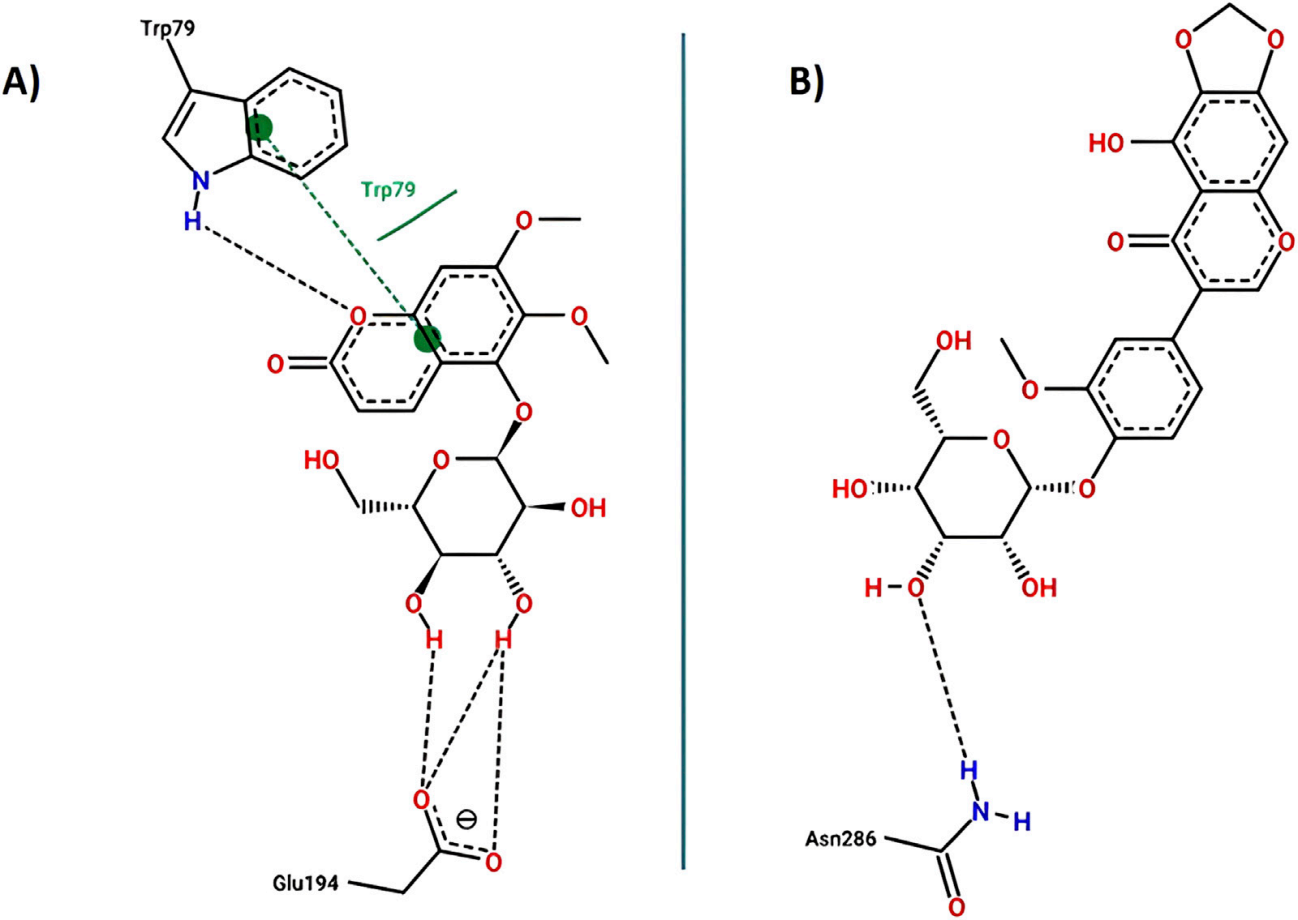
Key interactions between **(A)** 5GDC; **(B)** irifloside with butyrylcholinesterase.

Further analysis of the LBE and key interactions reveals interesting insights into the stability of the complexes. The comparison of interactions after docking and after 100 ns of simulation reveals that all of the key interactions were lost, and another were formed, increasing or decreasing the stability of protein-ligand complexes. While all three compounds form stable complexes with BChE, the binding of irifloside is particularly noteworthy due to its strong interaction with key residues in the enzyme’s active site. Figure 13B highlights that irifloside forms stable interactions with His259 and His94, contributing to the strong binding affinity, the aromatic core of irifloside interacts with additional residues in the active site, such as His259 and His94, further stabilizing the complex. Figure 13B also demonstrates the importance of Trp227 and the aromatic interactions between irifloside and BChE, reinforcing the strong affinity of irifloside for the enzyme. These interactions, particularly the hydrogen bonding and π-π interactions, explain why irifloside exhibits a higher binding affinity compared to 5C3M and 5GDC, as observed in both molecular docking and molecular dynamics studies. These findings help clarify the role of key residues in the enzyme’s active site, particularly in the case of irifloside, where its interactions with essential residues play a significant role in its high binding affinity. Additionally, the specific binding mode of irifloside with BChE, including the stabilization provided by interactions with His259 and His94, is clearly depicted in Figure 13A, which visually supports the detailed interaction analysis discussed above.

#### 3.5.3 Tyrosinase molecular dynamics results

Dynamics simulations reveal the strong potential of 5GDC and irifloside to interact with the enzyme, forming stable protein-ligand complexes throughout the 100 ns simulation. Analysis of the RMSD and Rg reveals distinct behaviors for the two ligands. The 5GDC complex exhibits consistently lower RMSD and Rg values compared to the irifloside complex, indicating greater stability and compactness of the tyrosinase structure when bound to 5GDC. Figure 9C shows that the RMSD for 5GDC remains stable with a smaller increase over time, reflecting a strong and stable binding. The lower Rg value observed for the 5GDC complex suggests a more compact conformation of tyrosinase upon 5GDC binding. This increased compactness likely contributes to the higher LBE of 18.381 observed for 5GDC compared with Irifloside (0.191) and 5C3M (−209.739). The specific interactions between 5GDC and active site residues of tyrosinase likely drive this compaction.

**FIGURE 14:**
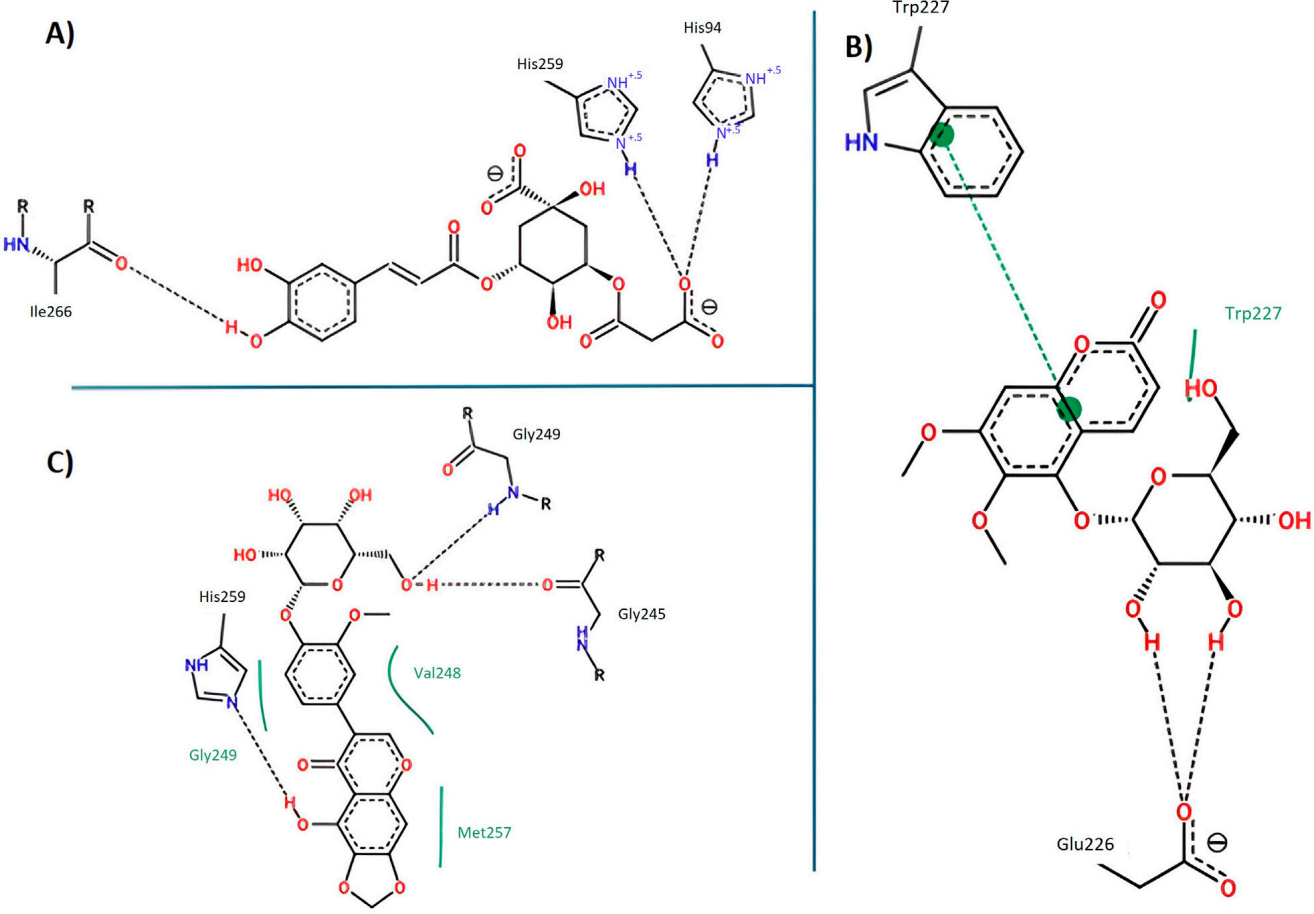
Key interactions between **(A)** 5C3M; **(B)** 5GDC; and **(C)** irifloside with tyrosinase.

The RMSF analysis identifies Leu75 and Leu77 as residues exhibiting the highest fluctuations. These residues may play a role in the observed changes in protein conformational dynamics and potentially influence the binding competitiveness of the ligands. Figure 10C shows that there are slight fluctuations for the 5C3M complex, indicating potential conformational changes or instability. That could be explained by the loss of hydrogen bonds of ligands with His85, Asn81, Asn 260, Ser 282 among others. Figure 11C shows that the overall Rg values for all complexes remain stable, suggesting that these conformational changes, if present, do not significantly alter the protein’s overall compactness. As shown in Figure 14A, the binding mode of 5GDC within the tyrosinase active site further illustrates the specific interactions that contribute to the stability and compactness of the complex. Further stabilizing interactions between 5GDC and Tyrosinase are highlighted in Figure 14B, which illustrates a key π-π interaction between the coumarin nucleus of 5GDC and Trp227, a new interaction formed after 100 ns of simulation. This interaction likely contributes significantly to the stabilization of the 5GDC-tyrosinase complex, supporting its strong binding affinity. Additionally, Figure 14B further depicts the complex’s stabilizing interactions, emphasizing the role of key residues in maintaining the overall structural integrity of the complex. These interactions ensure that the 5GDC-tyrosinase complex remains stable and contributes to the observed high binding affinity. Irifloside, in the data obtained from PLIP (data not shown), retains the interaction with His244 and forms new interactions with Gly245 and Gly249, His 259 and Asn260 after 100 ns of simulation, while in Figure 14C, it is shown hydrogen bonds with Gly245, Gly249 and His259 which can explain the stability of protein - ligand complex and the positive value of LBE.

#### 3.5.4 Nrf2-Keap1 molecular dynamics results

The stability of the protein-ligand complexes formed between Nrf2-Keap1 and 5C3M, 5GDC, and irifloside was assessed through 100 ns of molecular dynamics simulations. While the RMSD values initially appear relatively constant, a noticeable increase is observed starting at approximately 50 ns. This increase, exceeding 3 Å by 100 ns, suggests a progressive destabilization of the complexes. Figure 9D shows that the RMSD for the complexes increases progressively over time, indicating a loss of stability in the protein-ligand interactions, particularly in the 5C3M, 5GDC, and irifloside complexes. A similar trend is observed in the Rg plots, indicating that these complexes undergo significant changes in compactness over the course of the simulation. Figure 10D reveals substantial fluctuations in the RMSF profiles, especially at residues ALA292 and PRO293, suggesting that these residues play a crucial role in the observed destabilization. The concurrent increase in Rg with the RMSD suggests that the destabilization is accompanied by an expansion or unfolding of the protein structure. Figure 11D supports this interpretation by showing that the Rg values for these complexes increase progressively, further indicating that the protein structure undergoes a conformational change, likely leading to a less compact structure as a result of ligand binding.

**FIGURE 15:**
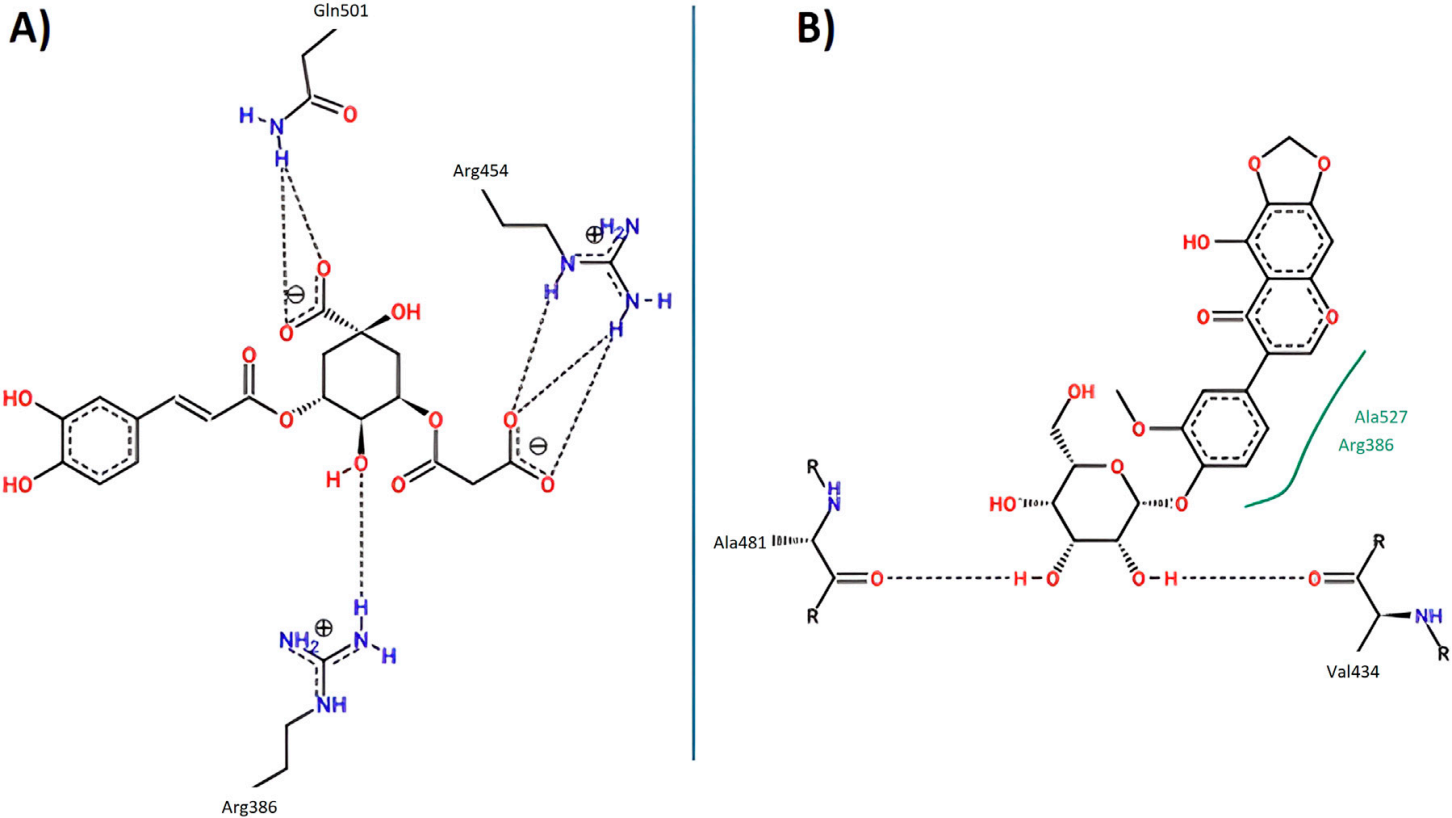
Key interactions between **(A)** 5C3M; **(B)** irifloside with Nrf2-Keap1 complex.

In addition to these observations, further analysis highlights the stabilization of irifloside binding within the active site of Nrf2-Keap1. Figure 15B reveals the critical interactions between irifloside and key residues such as Trp279 and Tyr130, which stabilize the complex. These hydrogen bonds and hydrophobic interactions are pivotal in maintaining the overall stability of the protein-ligand complex, despite the observed conformational changes in the protein structure. Figure 15B elaborates on the interactions within the peripheral anionic site (PAS), where the aromatic rings of irifloside interact strongly with Trp279, further enhancing the complex’s stability. Additionally, the role of Tyr130 and Trp279 in supporting irifloside’s binding, demonstrating that these stabilizing contacts help mitigate the destabilization observed in the protein structure. These interactions suggest that, while the Nrf2-Keap1 structure may undergo some localized conformational changes, the stabilizing interactions between the ligand and protein provide a strong foundation for the overall stability of the complex.

The flexibility observed in residues such as Ala292 and Pro293 in Figure 10D could potentially lead to disruptions in key interactions, yet the consistent hydrogen bonding and hydrophobic interactions observed in Figures 15A,B, underscore the robustness of irifloside’s binding, likely contributing to its high binding affinity despite these conformational adjustments. It is important to note that all the initial key interactions shown in protein - ligand complexes after docking, disappear after 100 ns of simulation.

The bioactive compounds identified in *L. quadripinnata* (5C3M, 5GDC, and irifloside) have demonstrated affinity for the Nrf2-Keap1 complex, which might facilitate the dissociation of Nrf2 from Keap1 and its subsequent translocation to the nucleus. This mechanism is associated with the activation of antioxidant and detoxification genes involved in mitigating oxidative stress. The ability of these compounds to modulate this pathway suggests a significant benefit in protecting cells under stress conditions. Moreover, in the case of cholinesterases (AChE and BChE), the compounds exhibit efficient interactions with the catalytic sites of these enzymes, particularly with key residues such as Ser200 and His440 as shown previously by other compounds (Kalita et al., 2025). This interaction may be linked to a reduction in enzymatic activity, enhancing the availability of acetylcholine. This indicates a potential positive effect on neurotransmission and cognitive function as demonstrated previously by Rivastigmine, a well-known dual inhibitor of brain cholinesterases (Korábečný et al., 2014). In this context, *L. quadripinnata* compounds stand out for their efficiency in activating antioxidant pathways and modulating the activity of key enzymes associated with neuroprotective processes, positioning them as promising candidates for further studies in neurodegenerative disease models. Supplementary Figure S7 illustrates the possible molecular interactions and stability of these compounds, highlighting their significant potential in the modulation of oxidative stress and neurodegenerative diseases.

## 4 Conclusion

The aqueous extract of *L. quadripinnata* contains a diverse array of secondary metabolites, primarily aromatic compounds, with high levels of phenolic constituents and notable antioxidant activity. These findings, along with theoretical analyses evaluating the inhibition of enzymes such as acetylcholinesterase, butyrylcholinesterase, and tyrosinase, as well as interactions with the Nrf2-Keap1 protein complex, support the potential of this fern as a promising source of bioactive molecules for the design of future studies related to oxidative stress. The theoretical approach, based on molecular docking and molecular dynamics simulations, allows the formulation of a hypothesis regarding a potential modulatory role in the antioxidant pathway. However, further experimental validation will be required through relevant *in vitro* and *in vivo* neurodegeneration models.

The computational strategy, which included validated molecular docking through redocking protocols, molecular dynamics simulations, and interaction profiling, enabled the formulation of hypotheses regarding potential modulatory roles of selected compounds in key antioxidant and neuroprotective pathways. Particularly, the consistent binding and interaction stability observed for irifloside and 5C3M across multiple targets strengthens the premise of their therapeutic relevance.

However, further experimental validation will be required through *in vitro* and *in vivo* neurodegeneration models to confirm the predicted activities and bioavailability. This study also highlights the integration of chromatographic, *in vitro*, and *in silico* methodologies as a comprehensive strategy for the exploration of natural products. In addition to contributing to the chemical characterization of *L. quadripinnata*, this work underscores the importance of combining multidisciplinary approaches to advance the identification and evaluation of the biological effects of bioactive compounds.

## Data Availability

The datasets presented in this study can be found in online repositories. The name of the repository and accession number can be found at: MetaboLights-MTBLS9336.
