# Supplementary material for: Biological potential of Lophosoria quadripinnata fern extract: integration of UHPLC/ESI/QToF/MS analysis, antioxidant activity, molecular docking and molecular dynamics simulation
Source: Front Pharmacol. 2025 Jul 11;16:1611733. doi: 10.3389/fphar.2025.1611733 (PMC12311474; doi:10.3389/fphar.2025.1611733)
Supplement: Supplementary file 1 [file DataSheet1.docx]

Supplementary figures


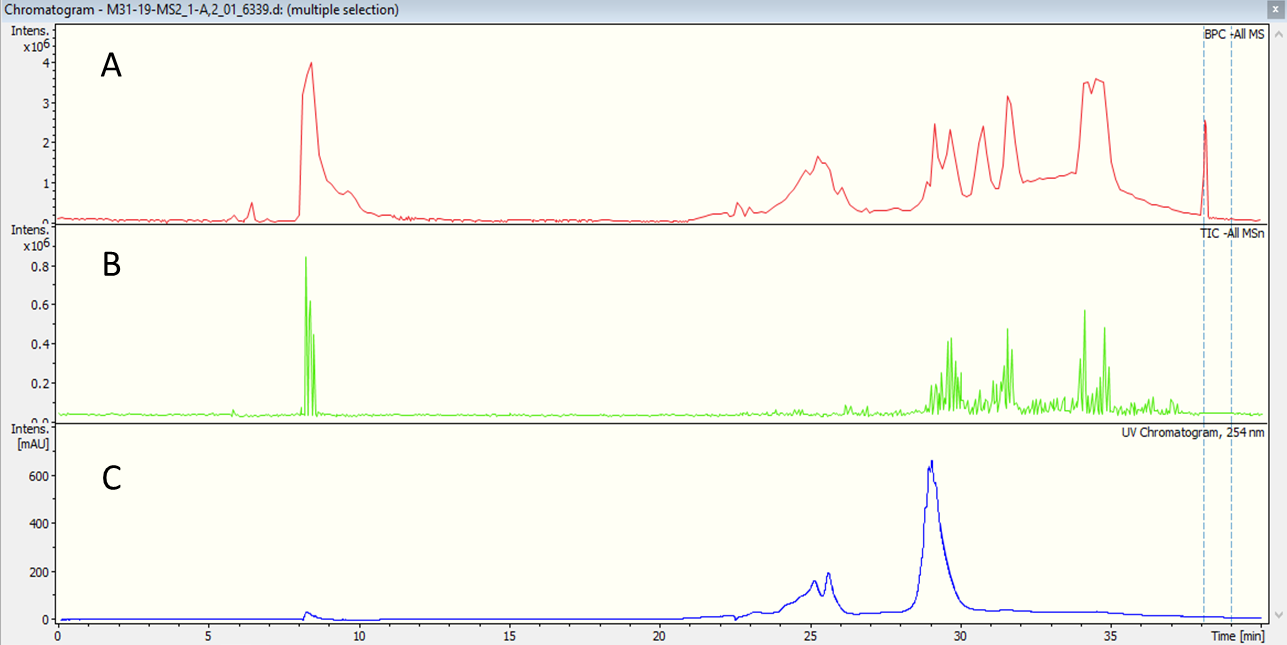


Supplementary figure 1. (A) Base peak chromatogram. (B) Total ion current TIC-All MSn chromatogram. (C) UV chromatogram 254 nm.


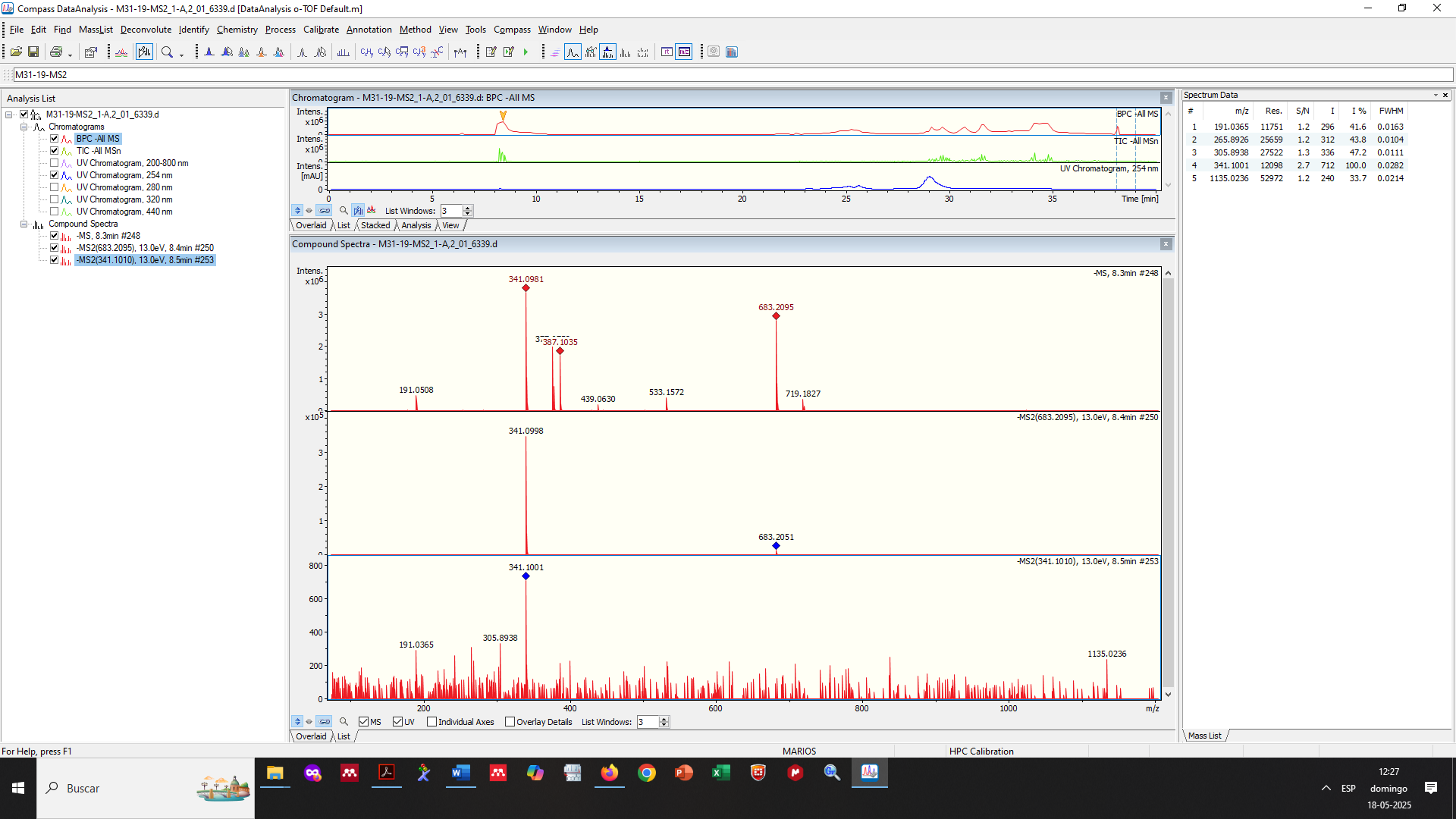


Supplementary figure 2. Full MS and MS spectra of peak 3.


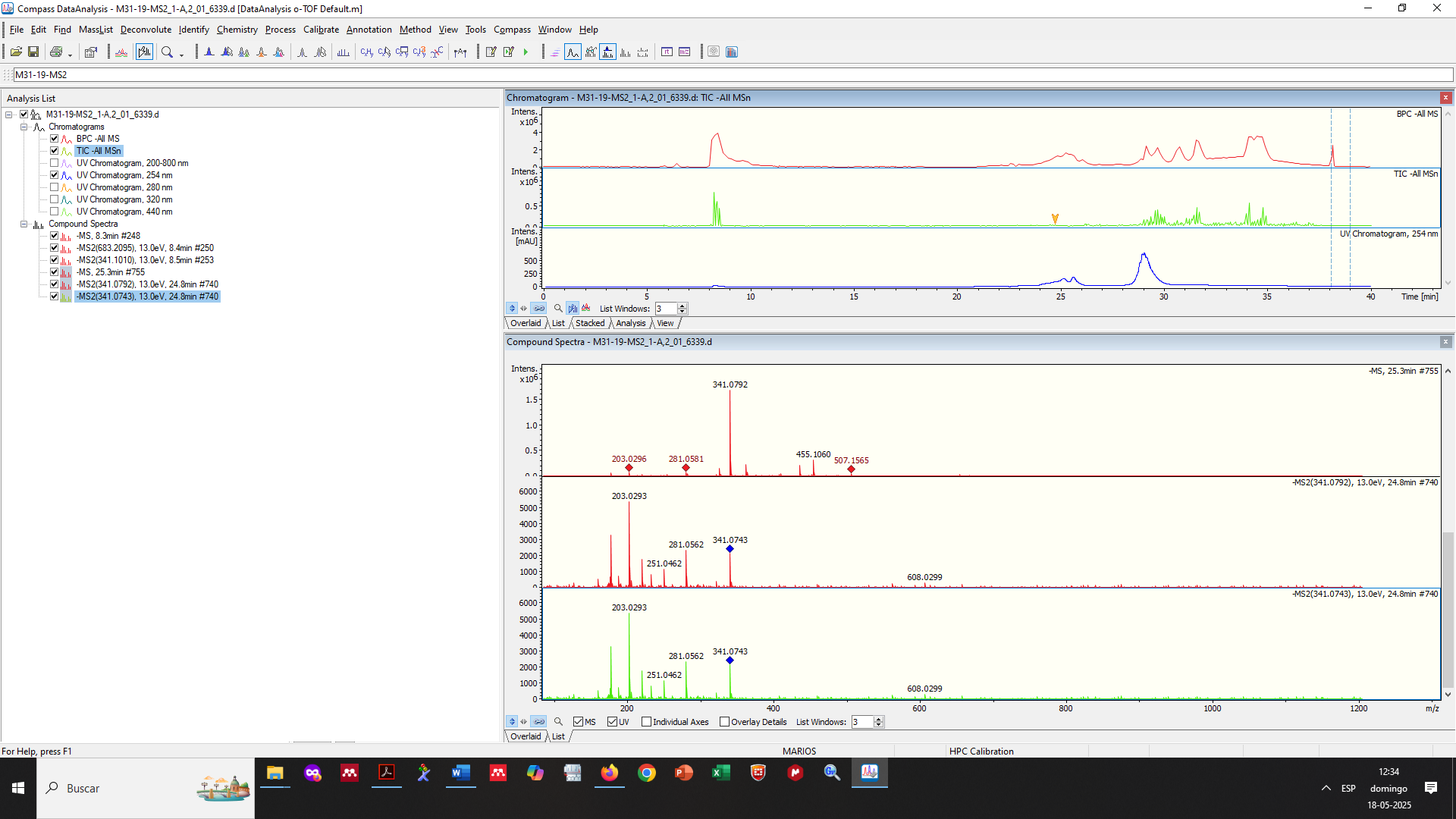


Supplementary figure 3. Full MS and MS spectra of peak 7.


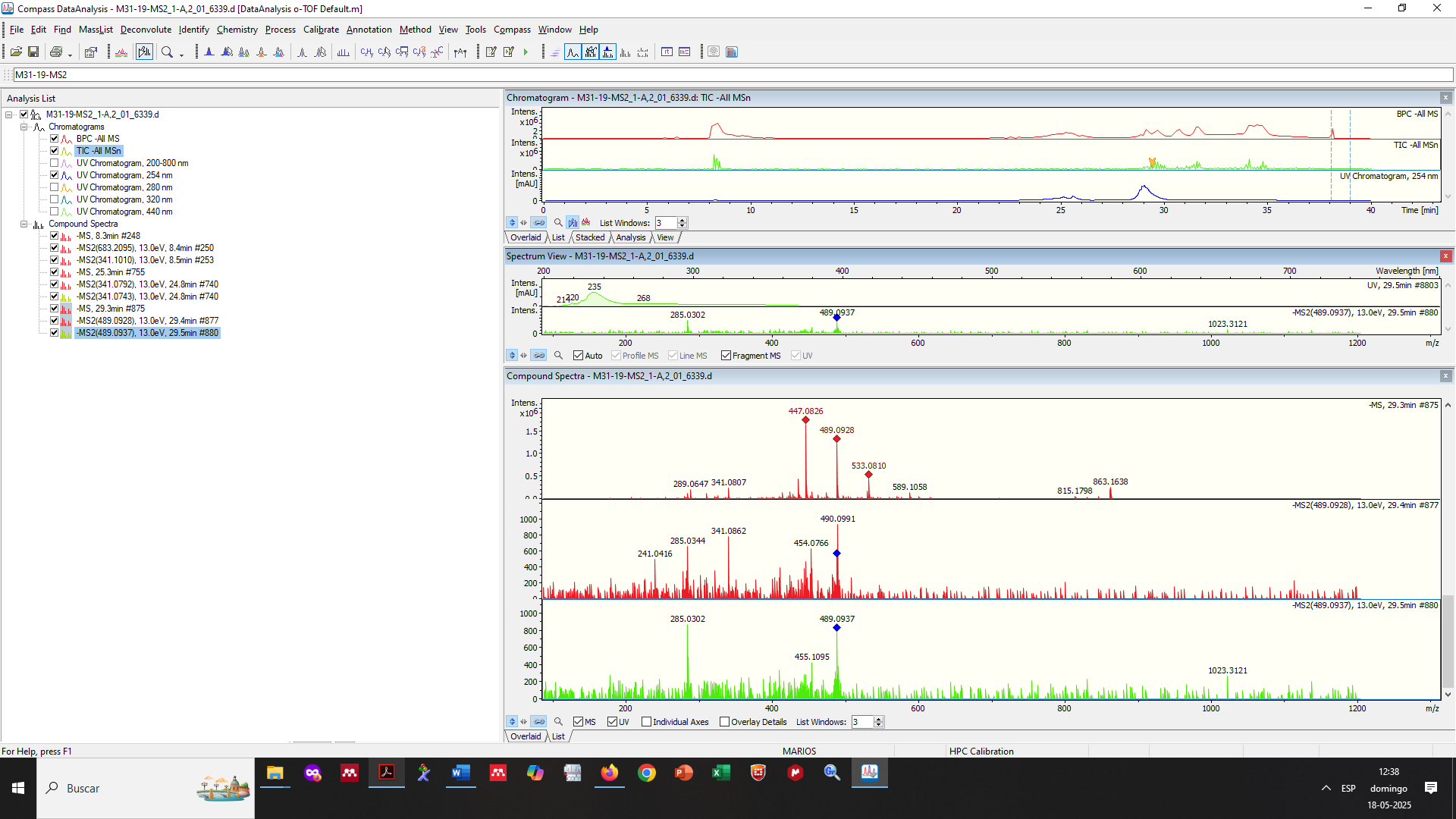


Supplementary figure 4. Full MS and MS spectra of peak 9.


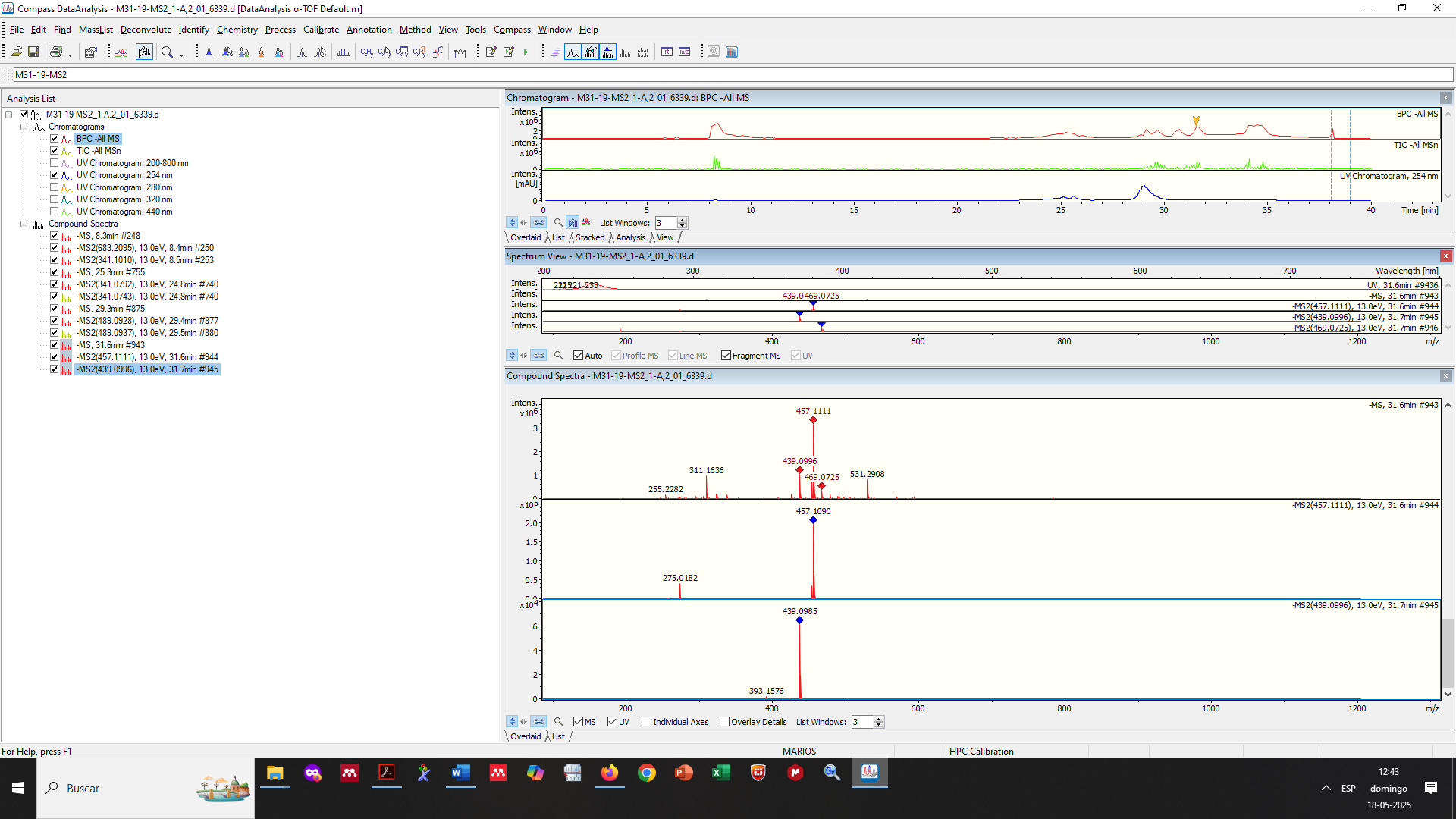


Supplementary figure 5. Full MS and MS spectra of peak 12.


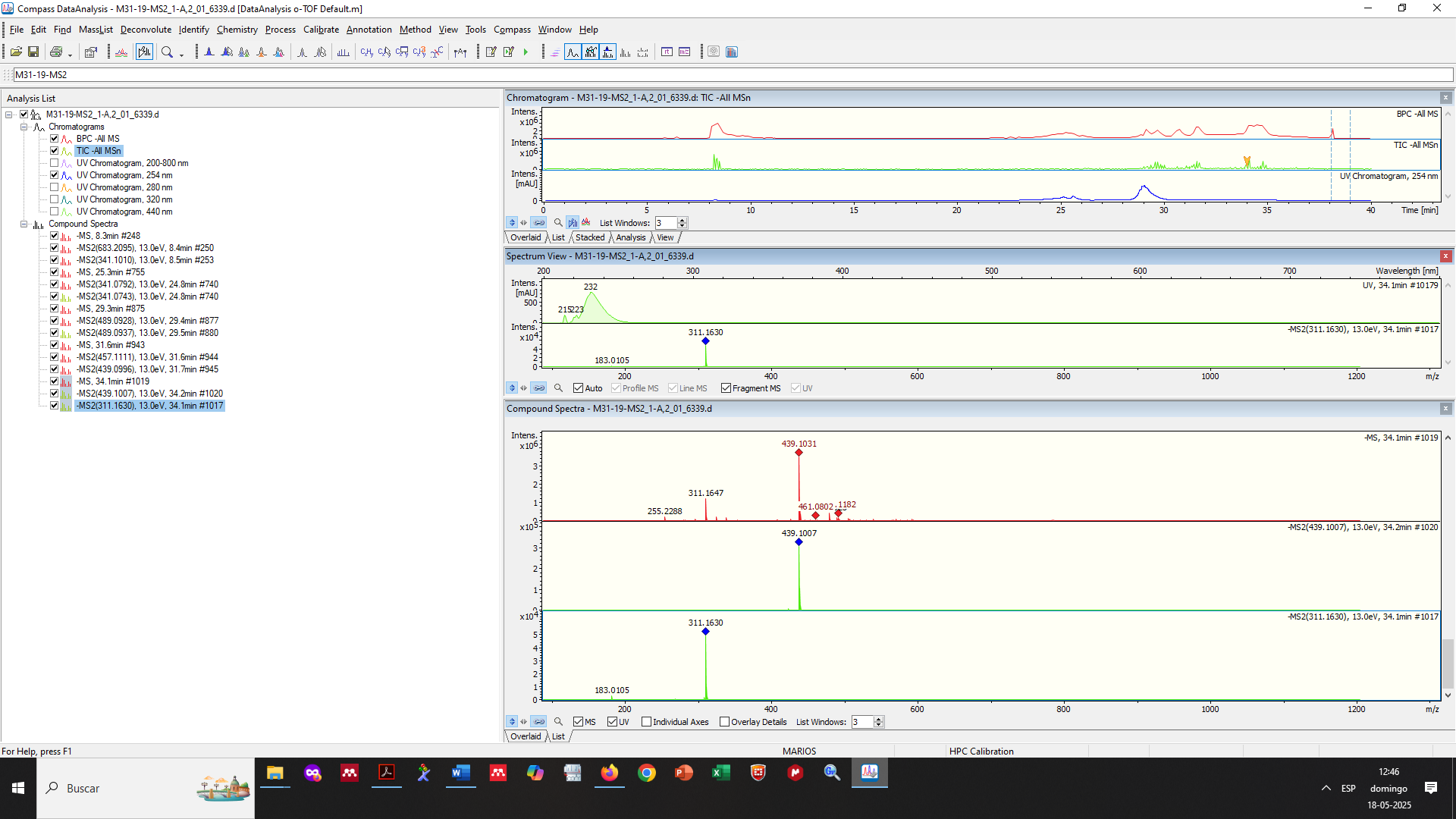


Supplementary figure 6. Full MS and MS spectra of peak 13.


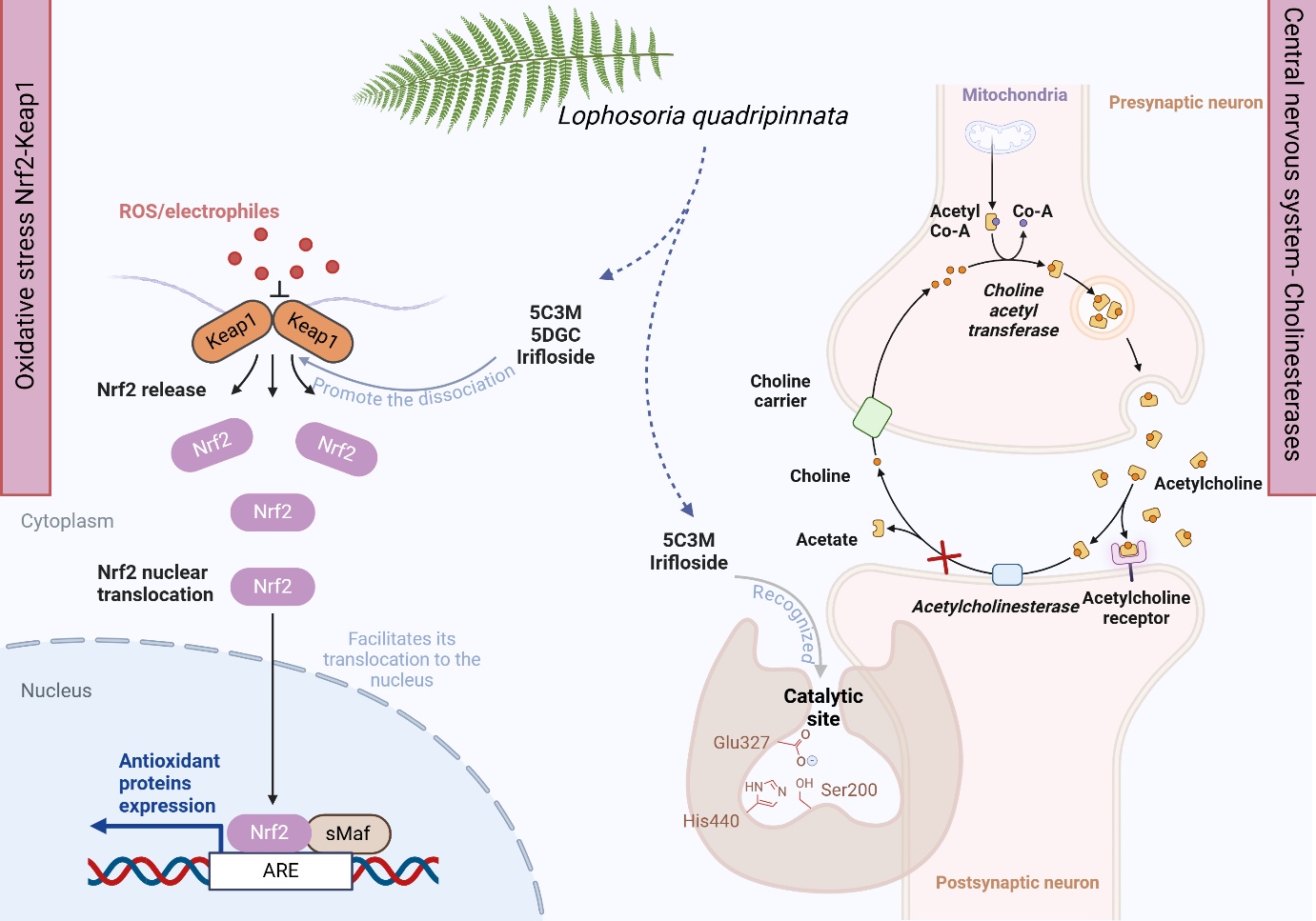


Supplementary figure 7. Proposed cellular mechanism of action in antioxidant and neuroprotective pathway modulation associated with *L. quadripinnata* compounds. Created in Biorender.co.
